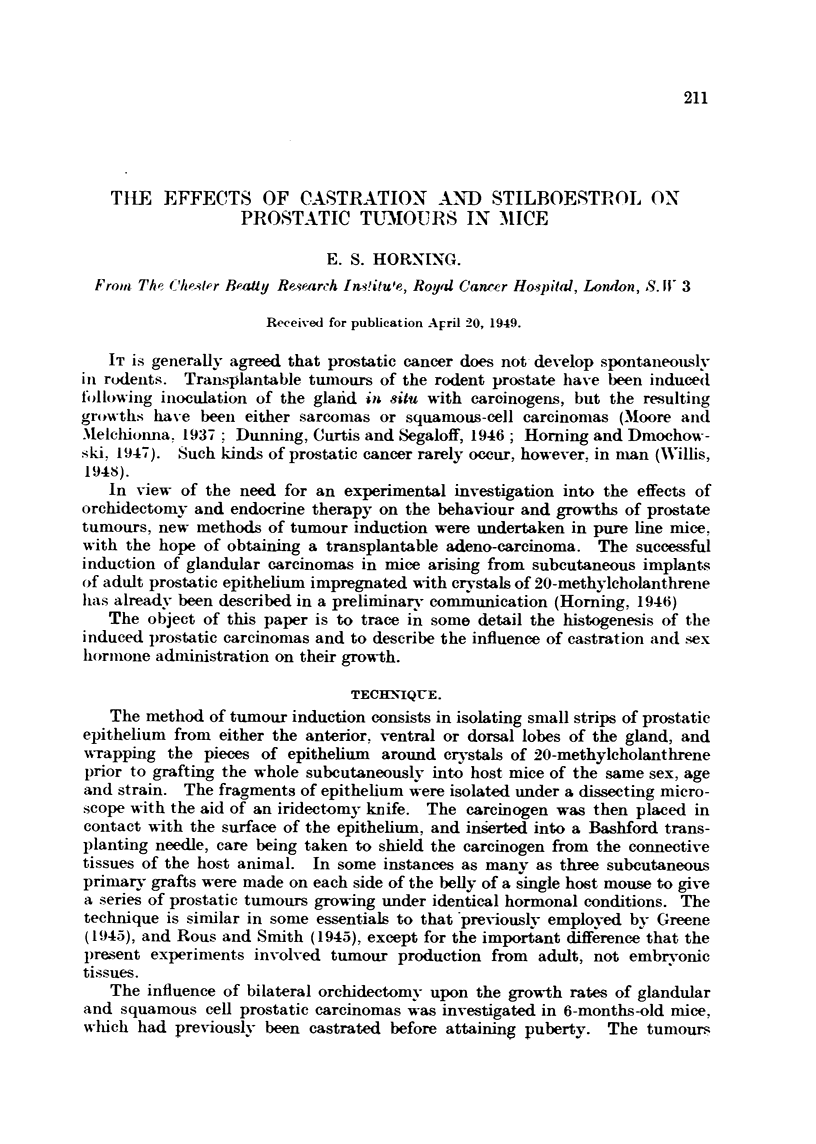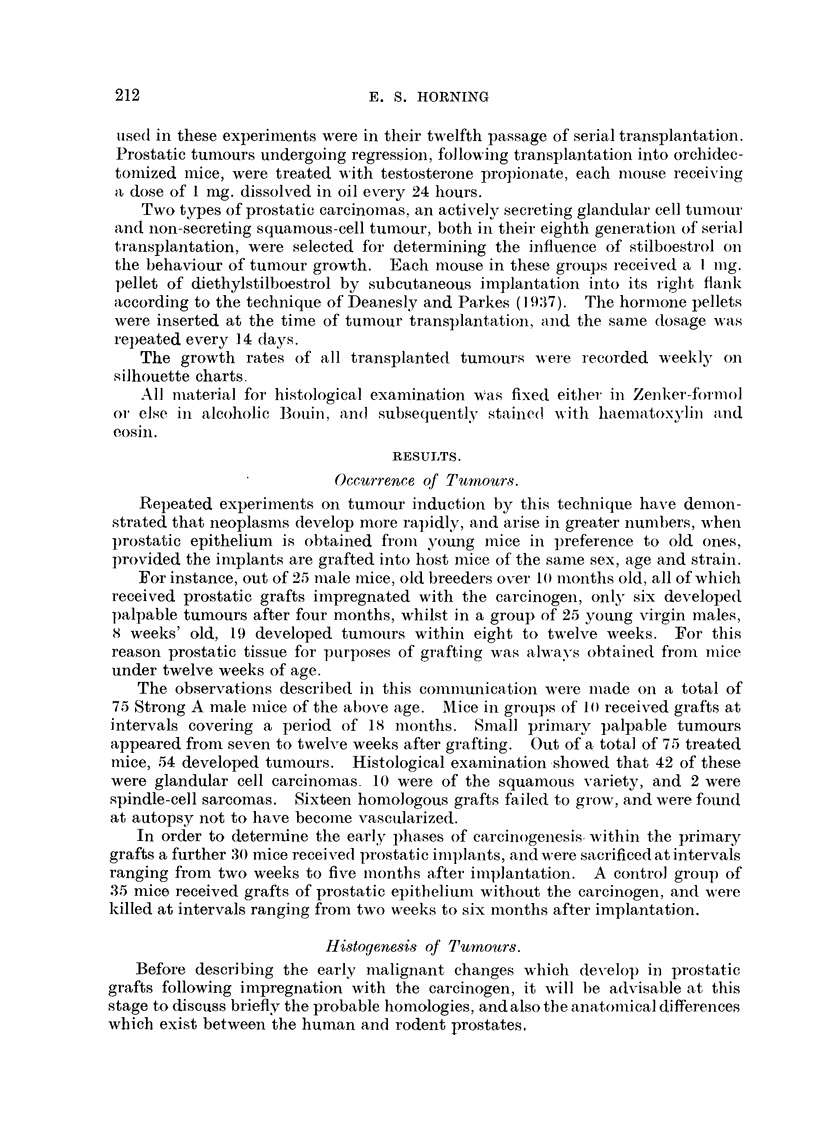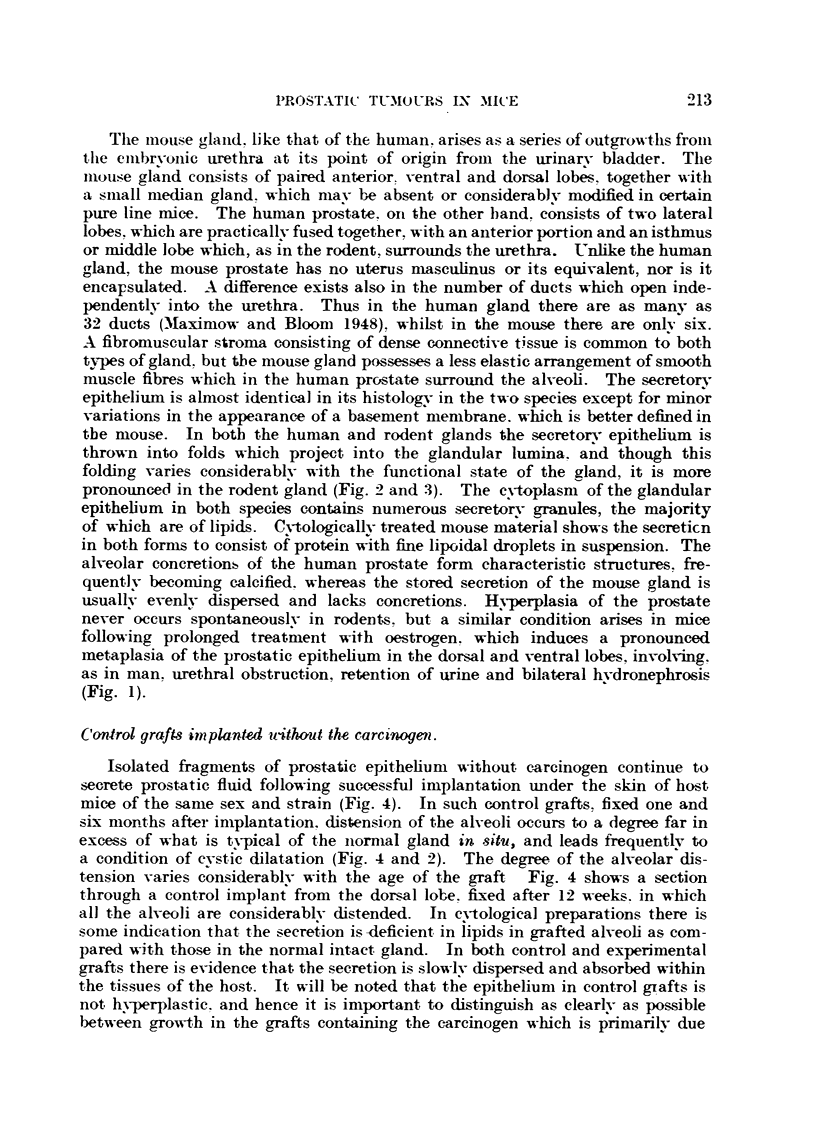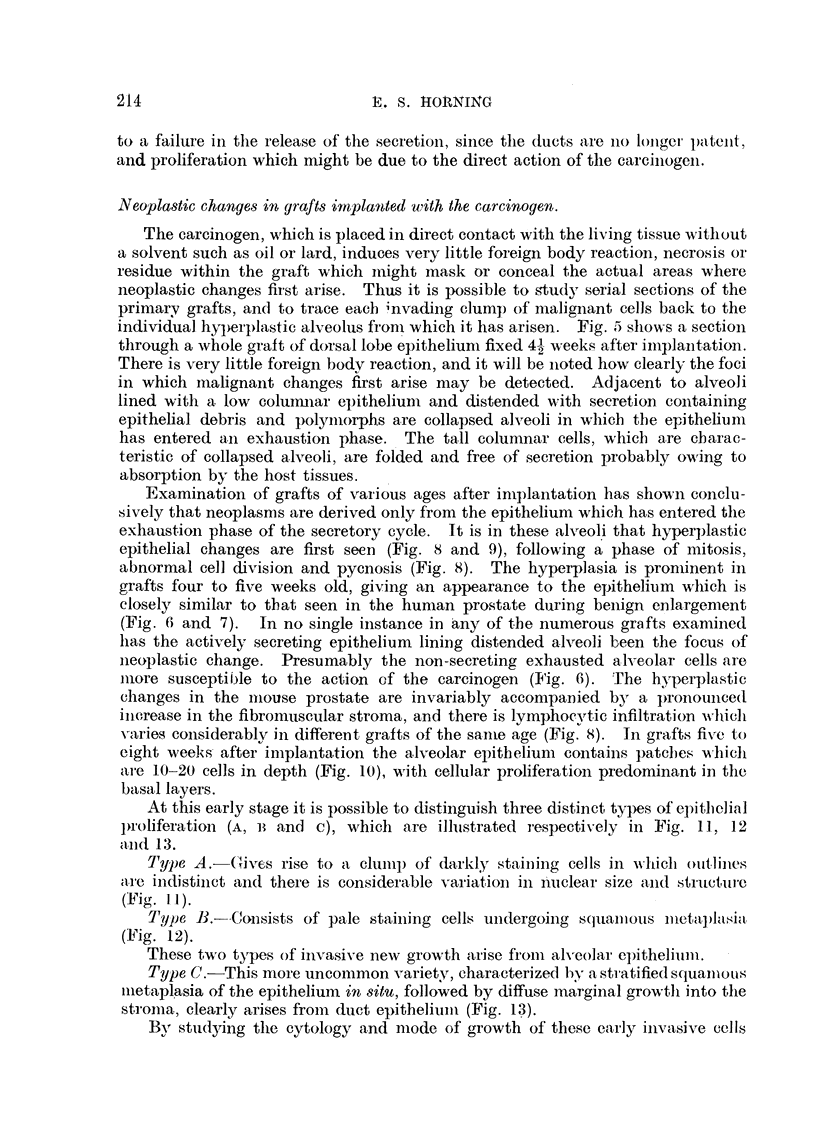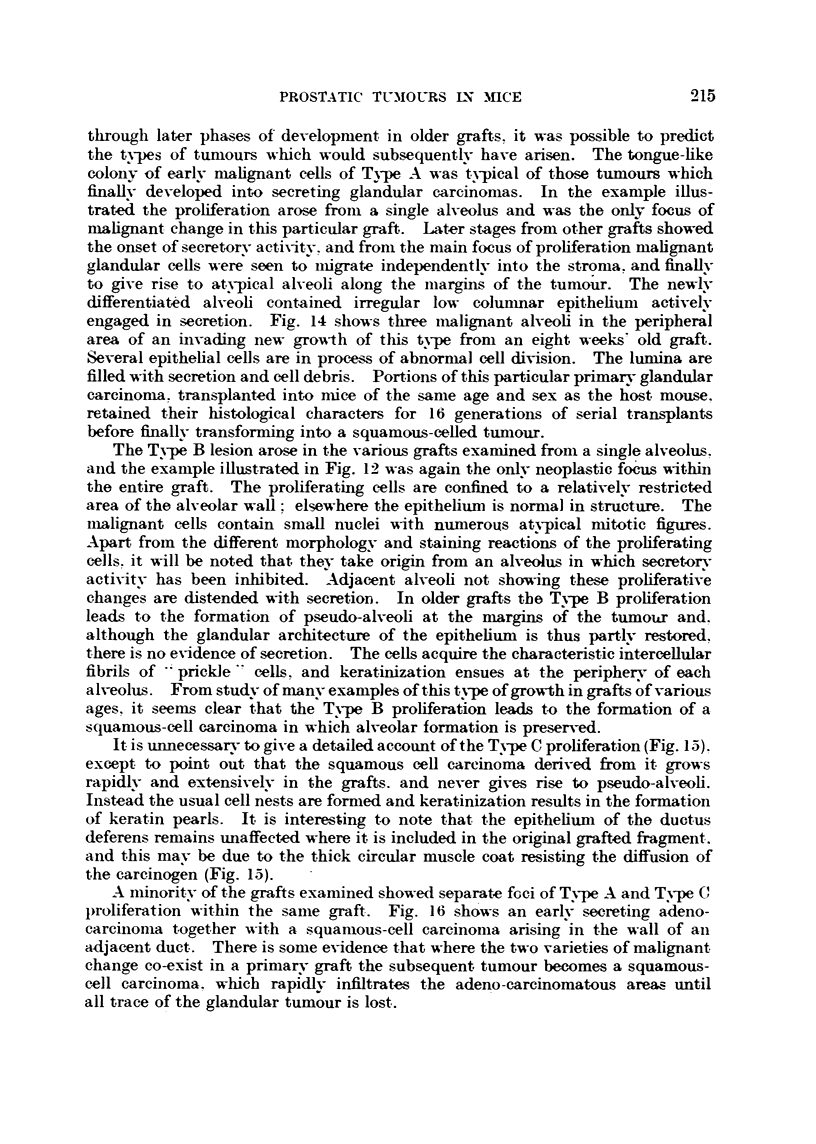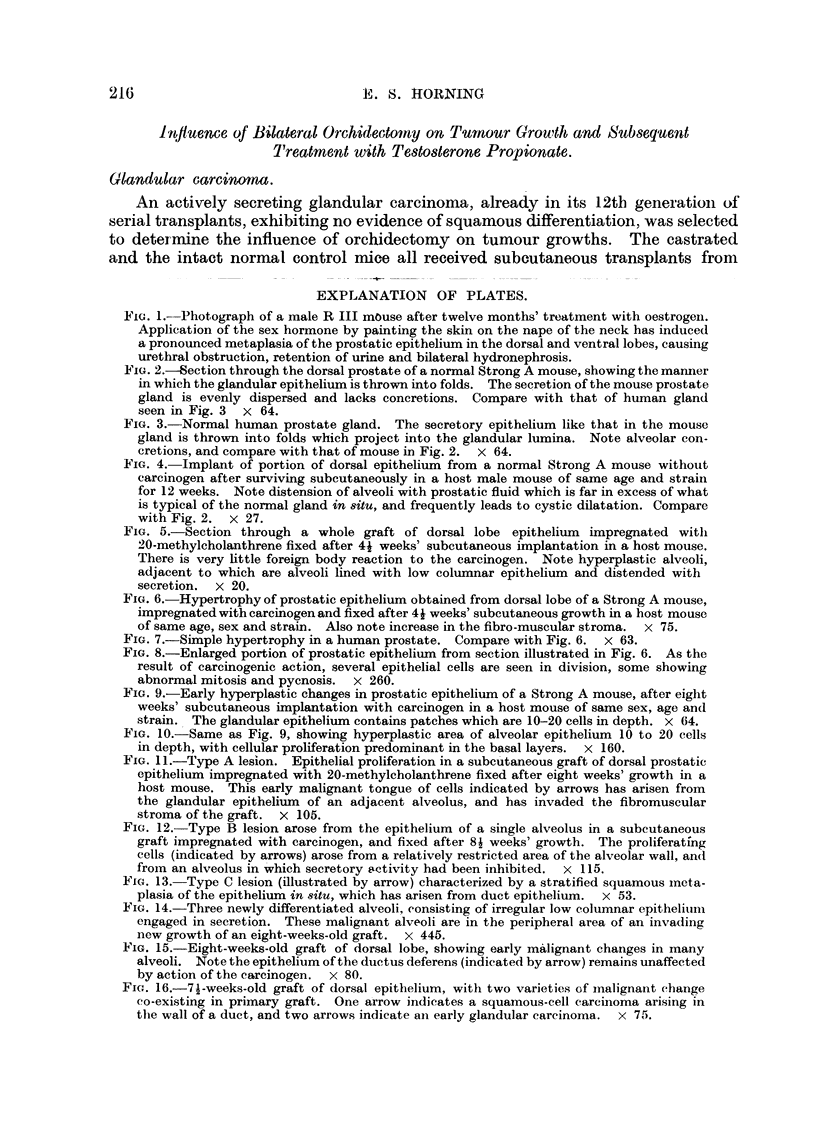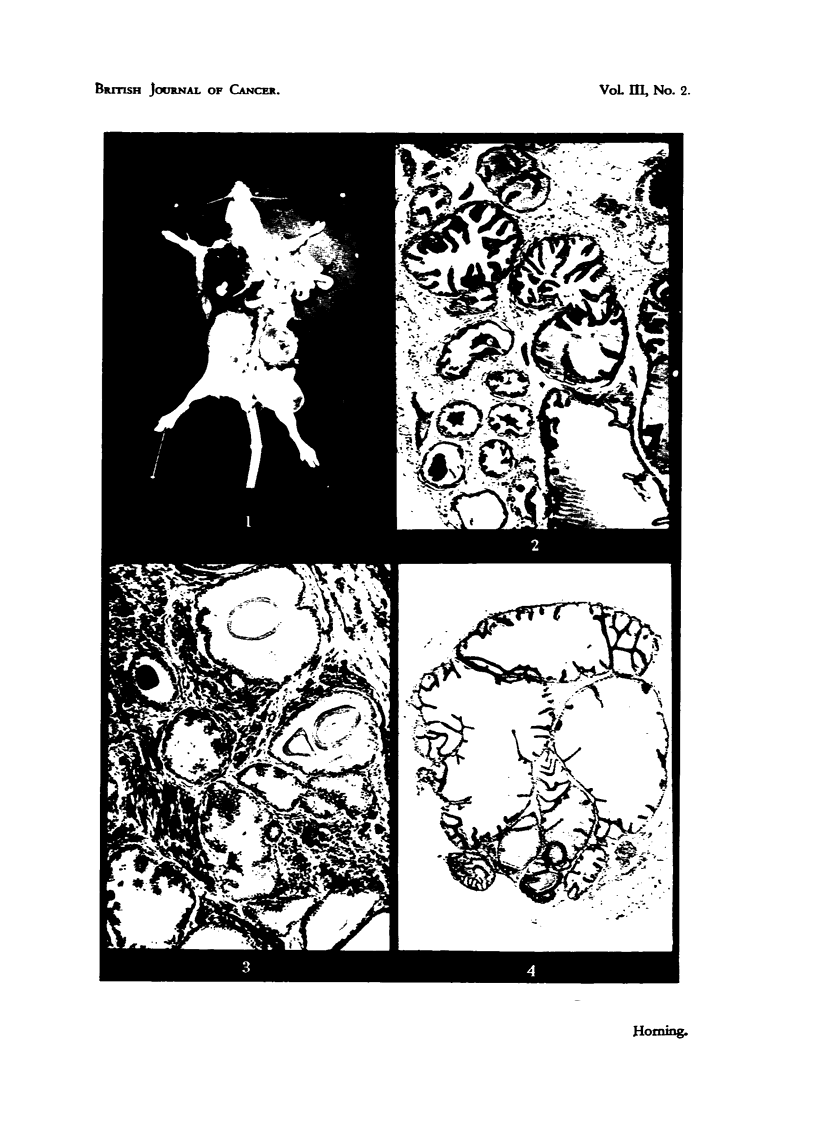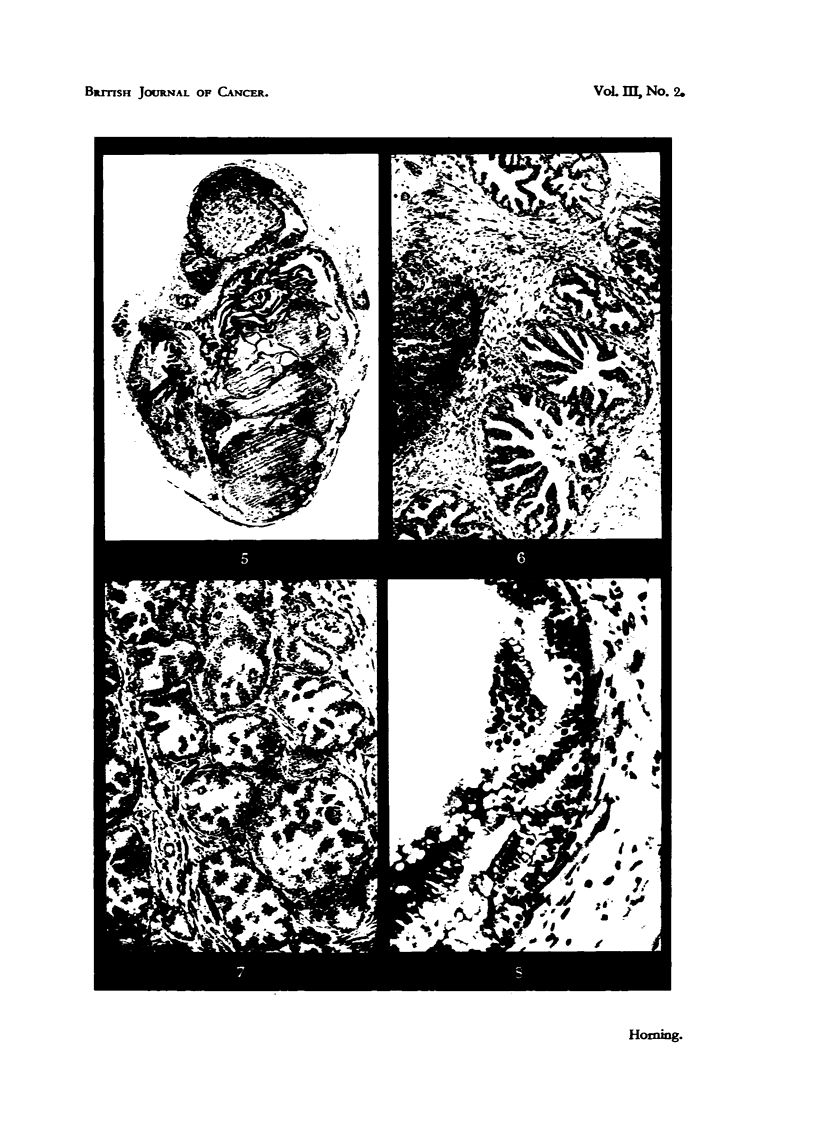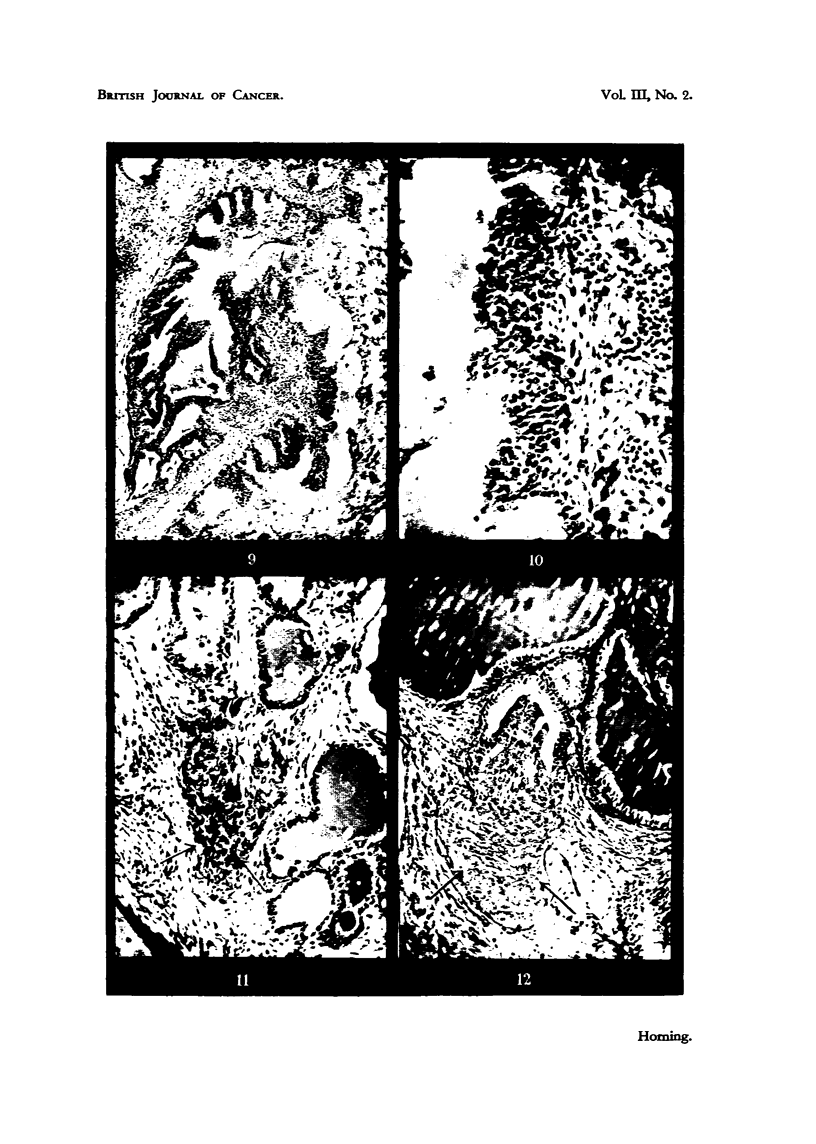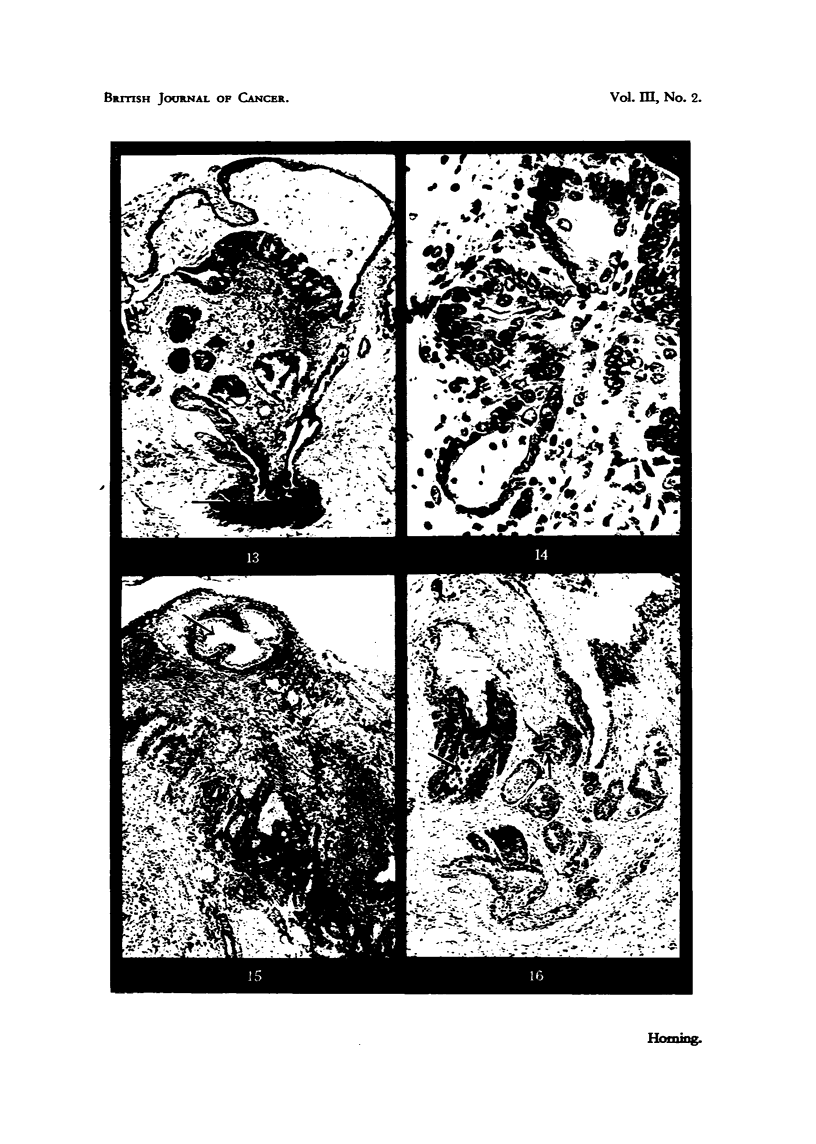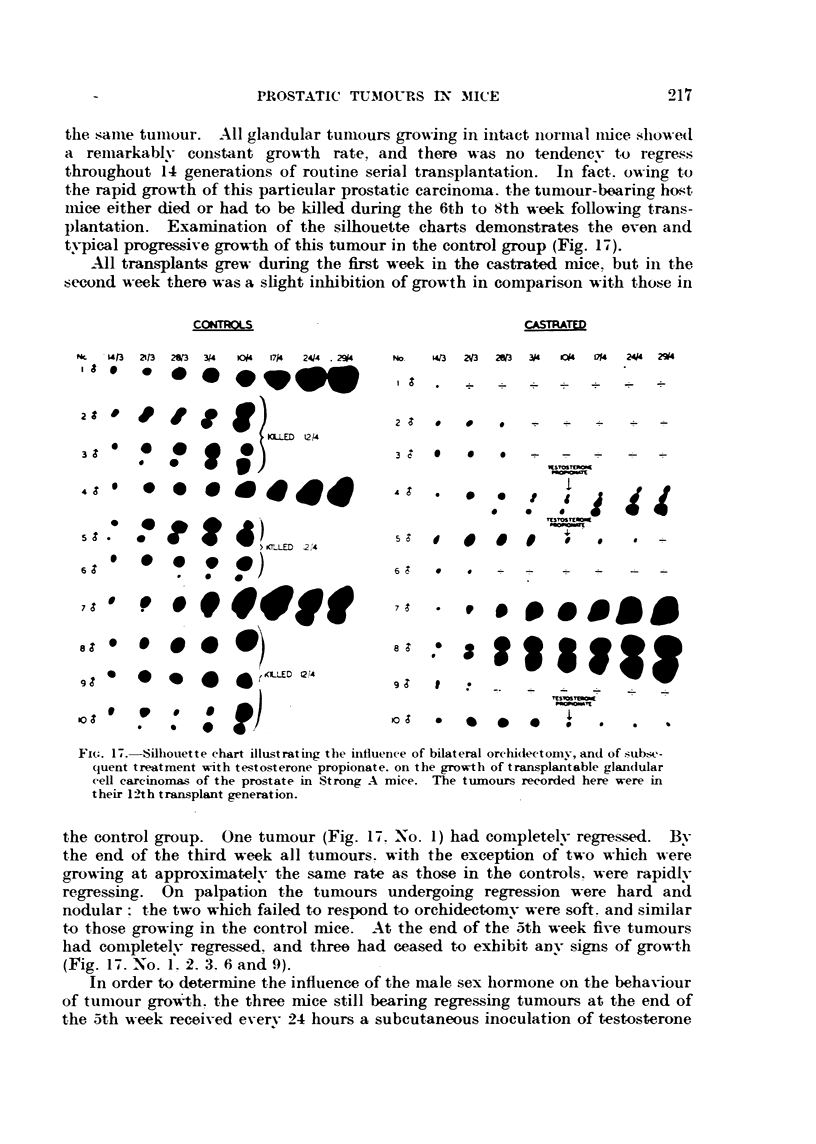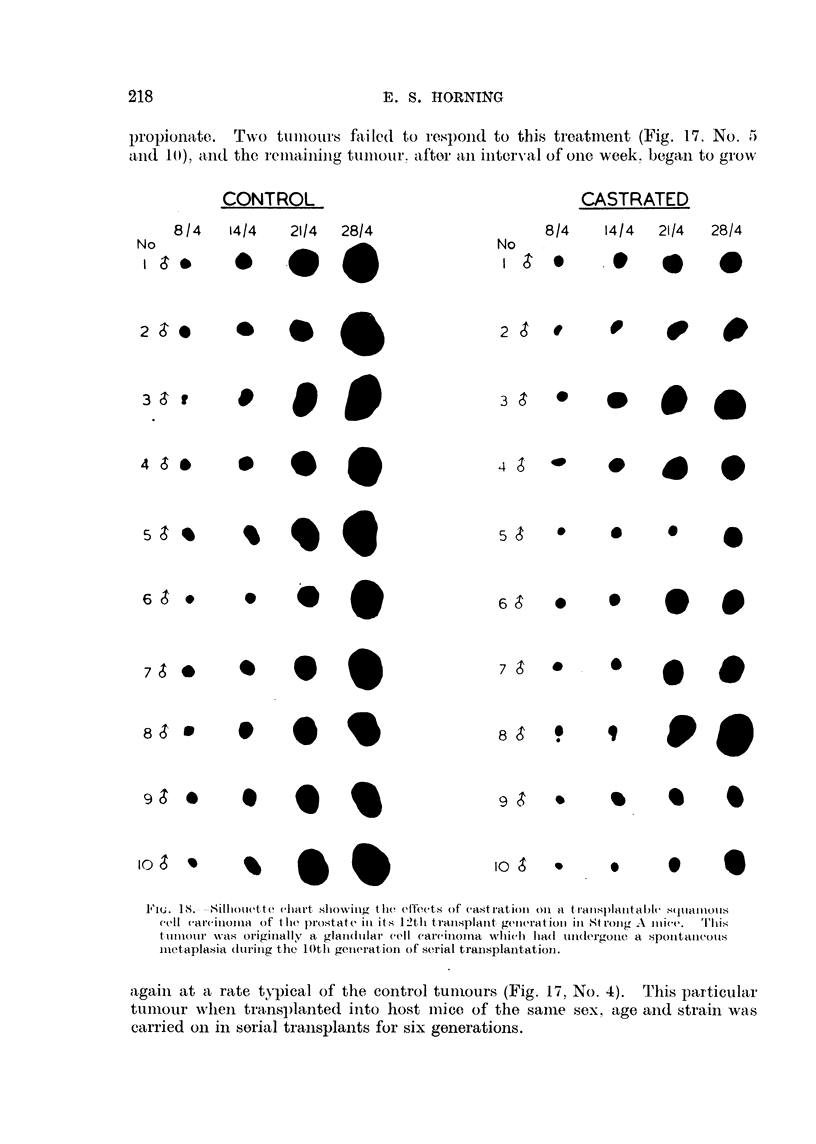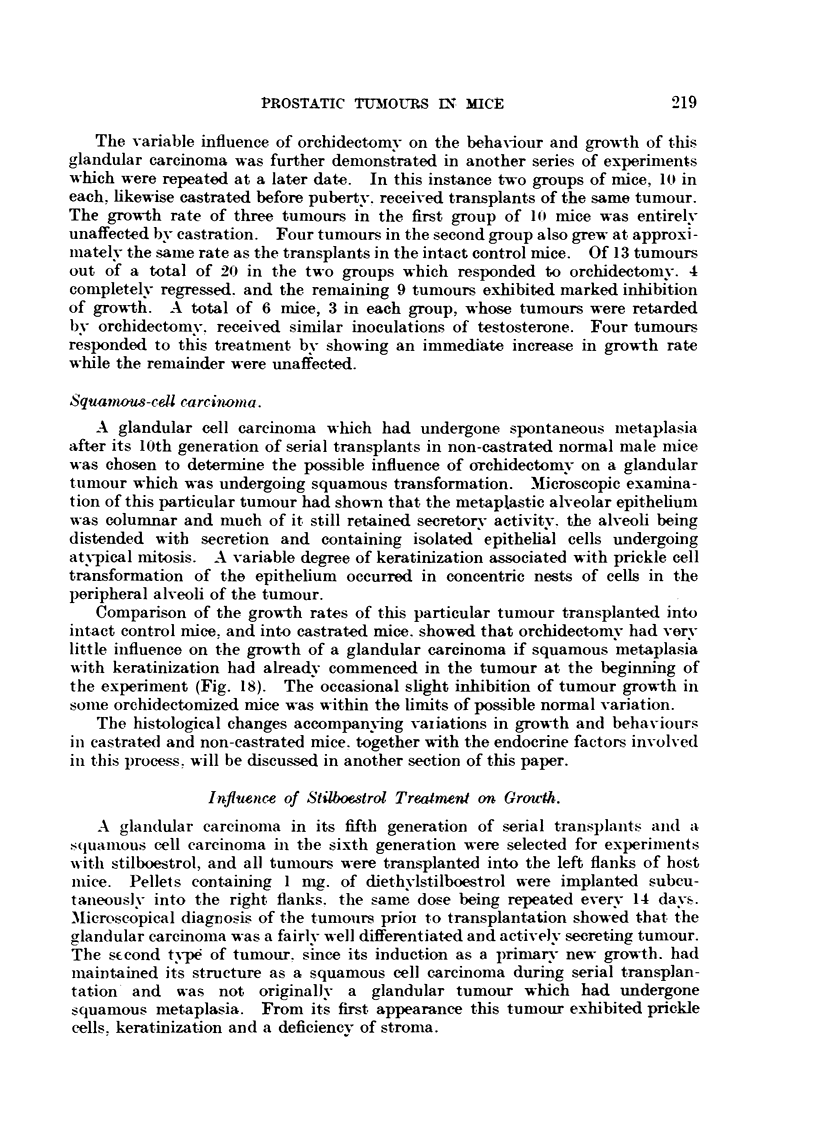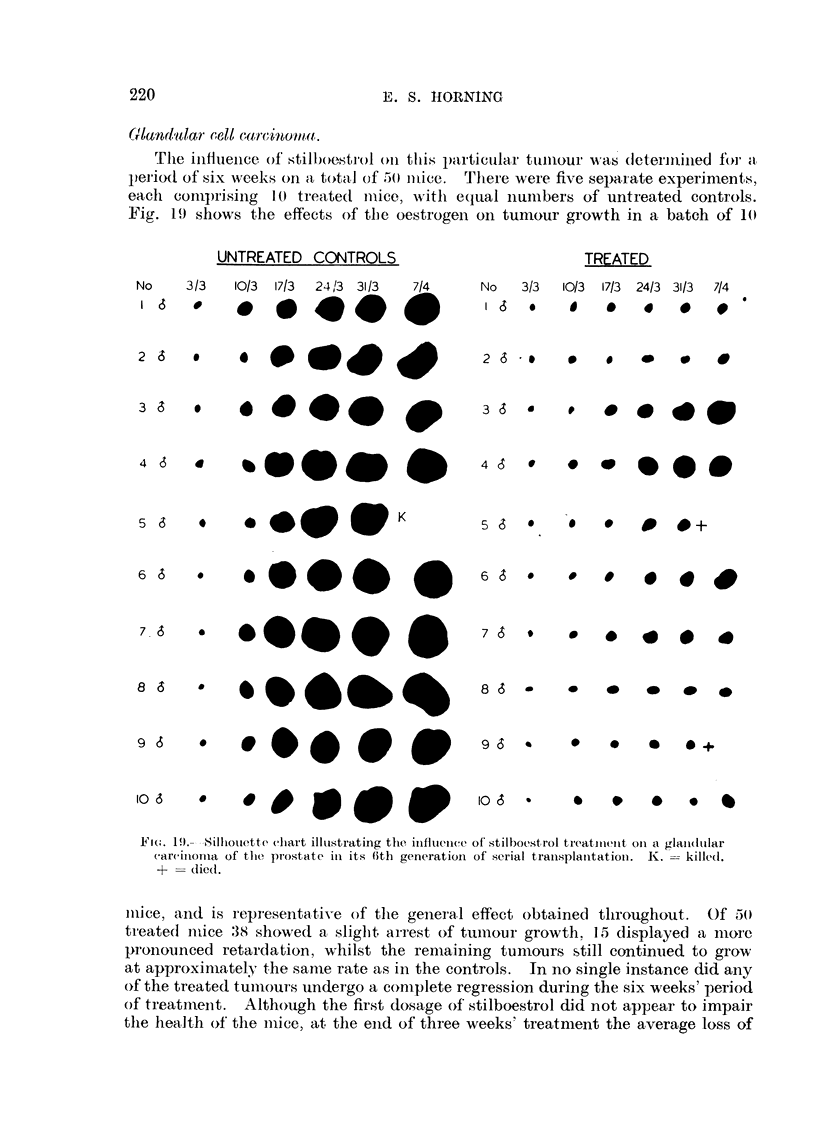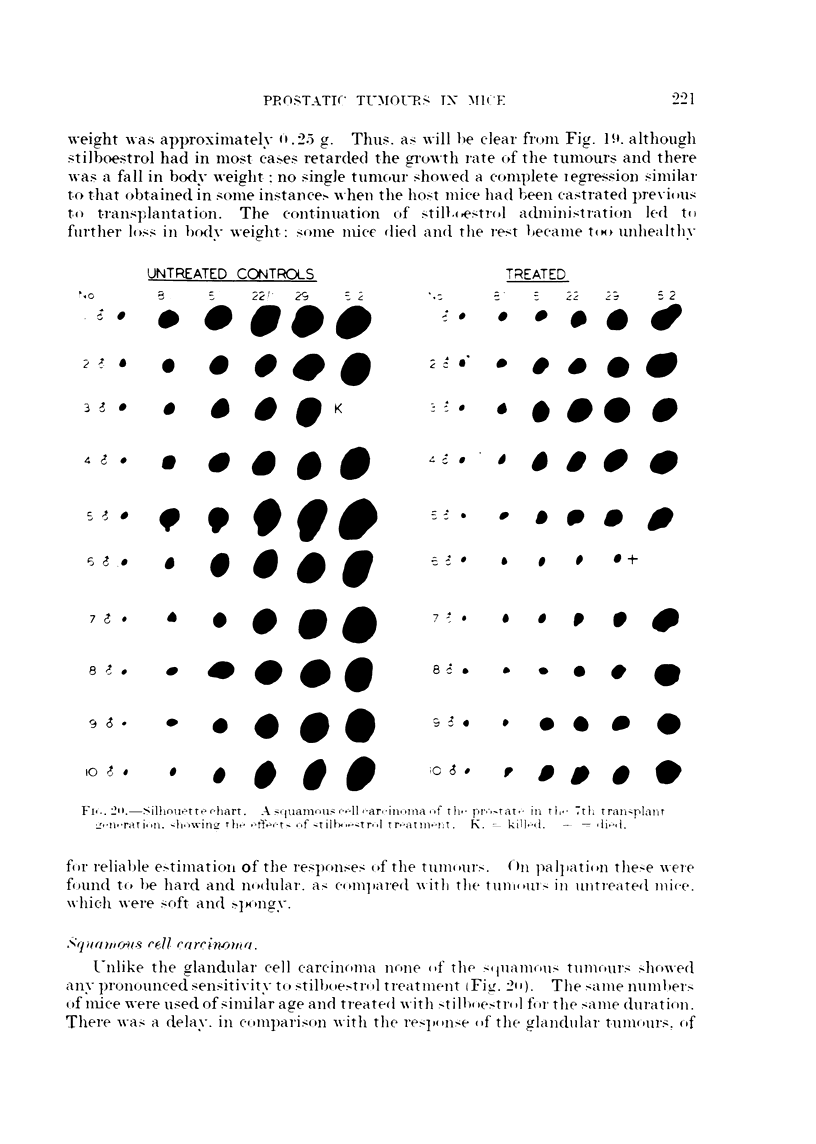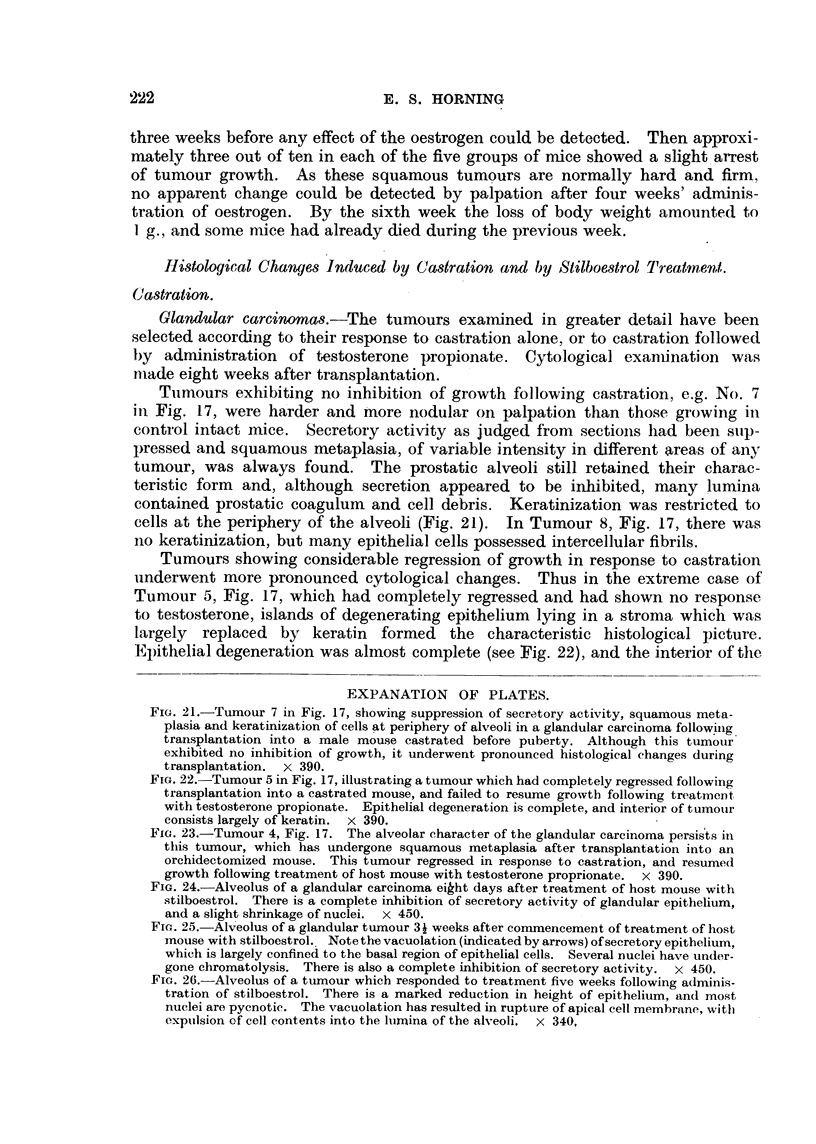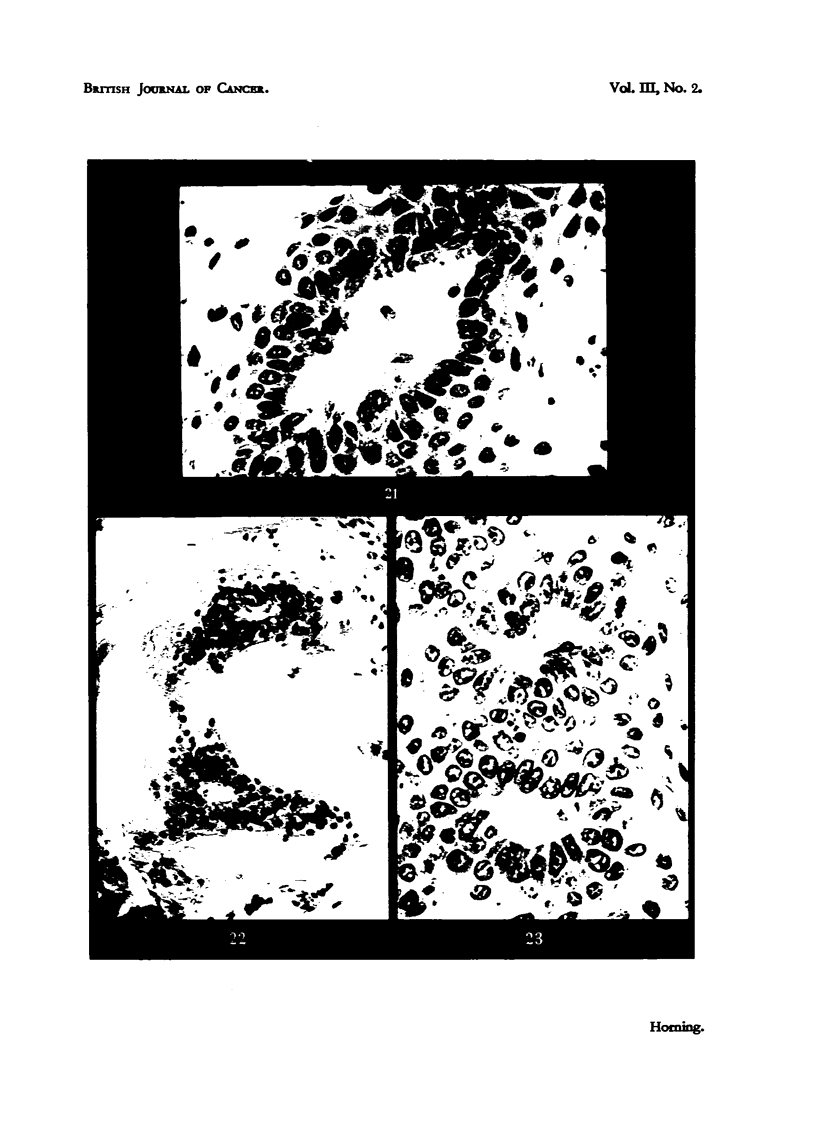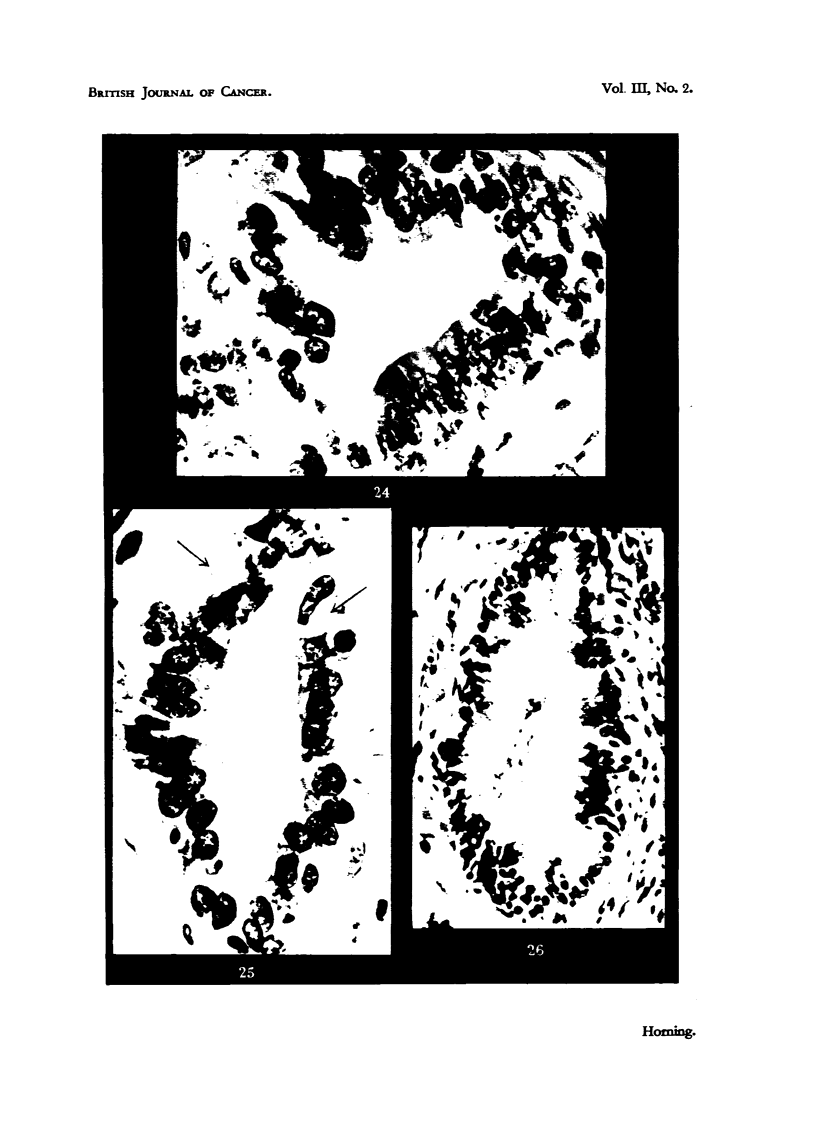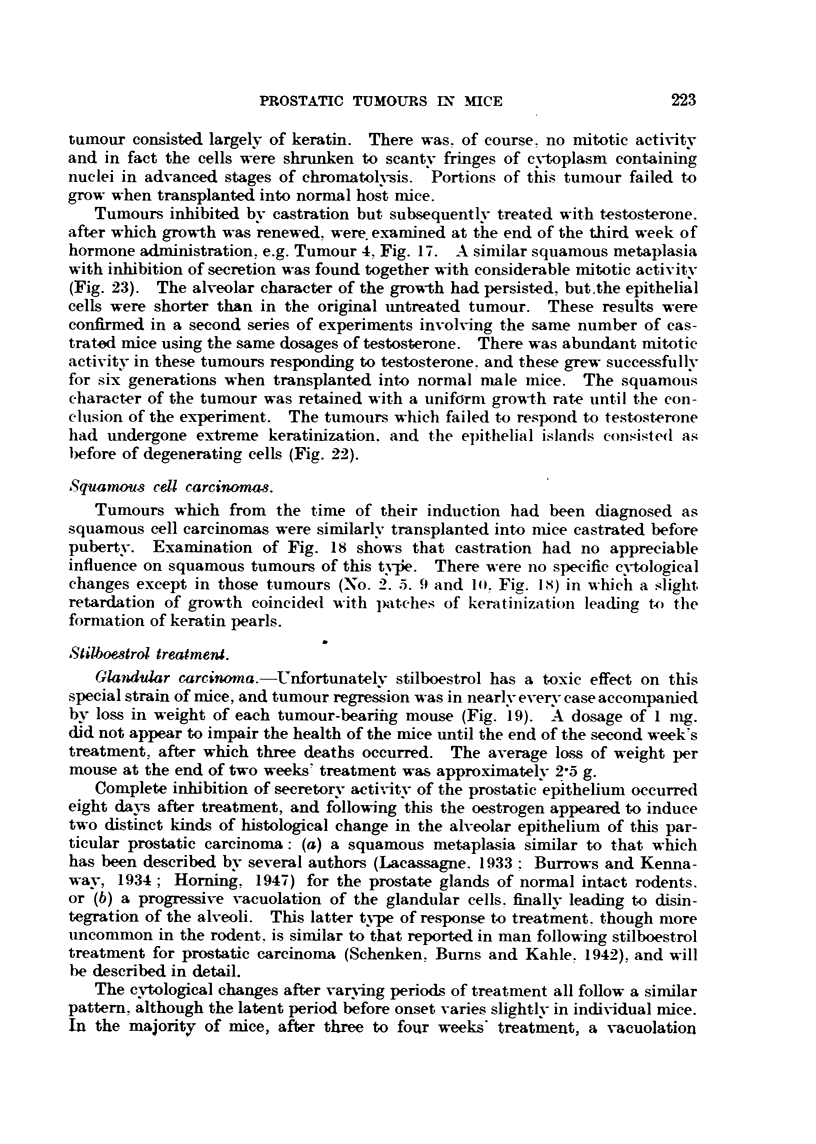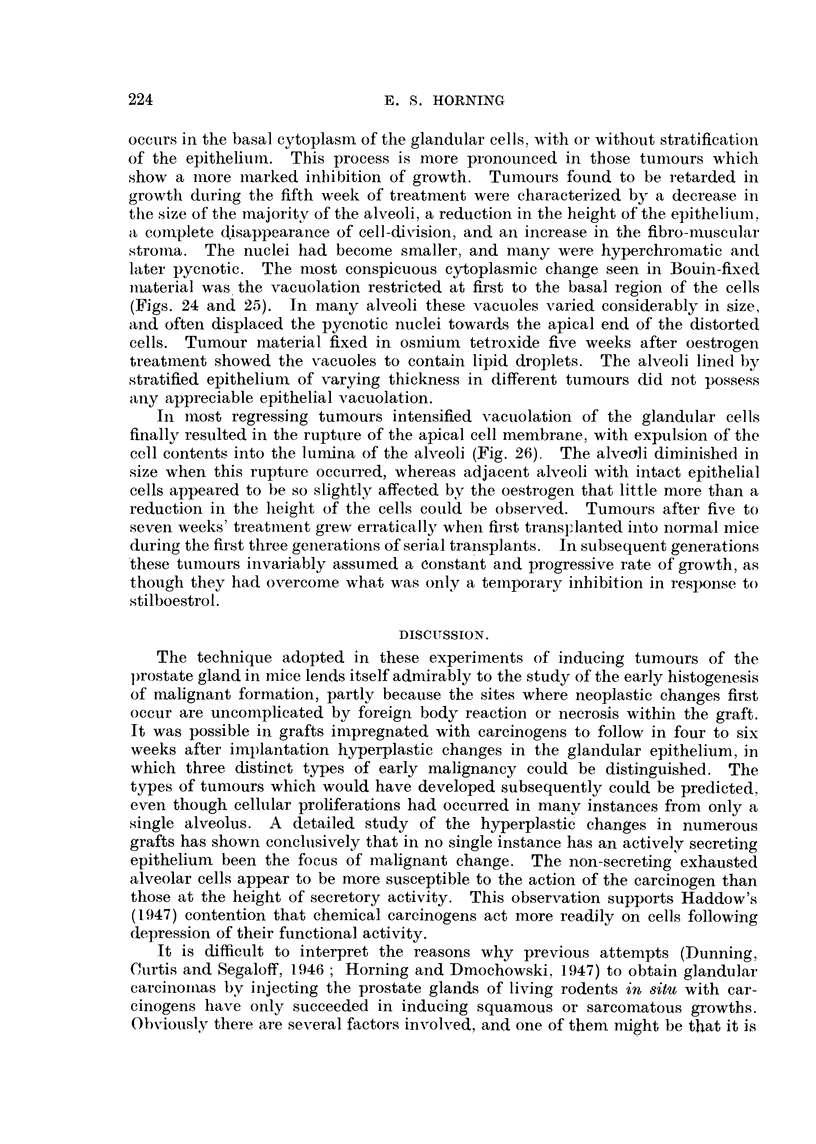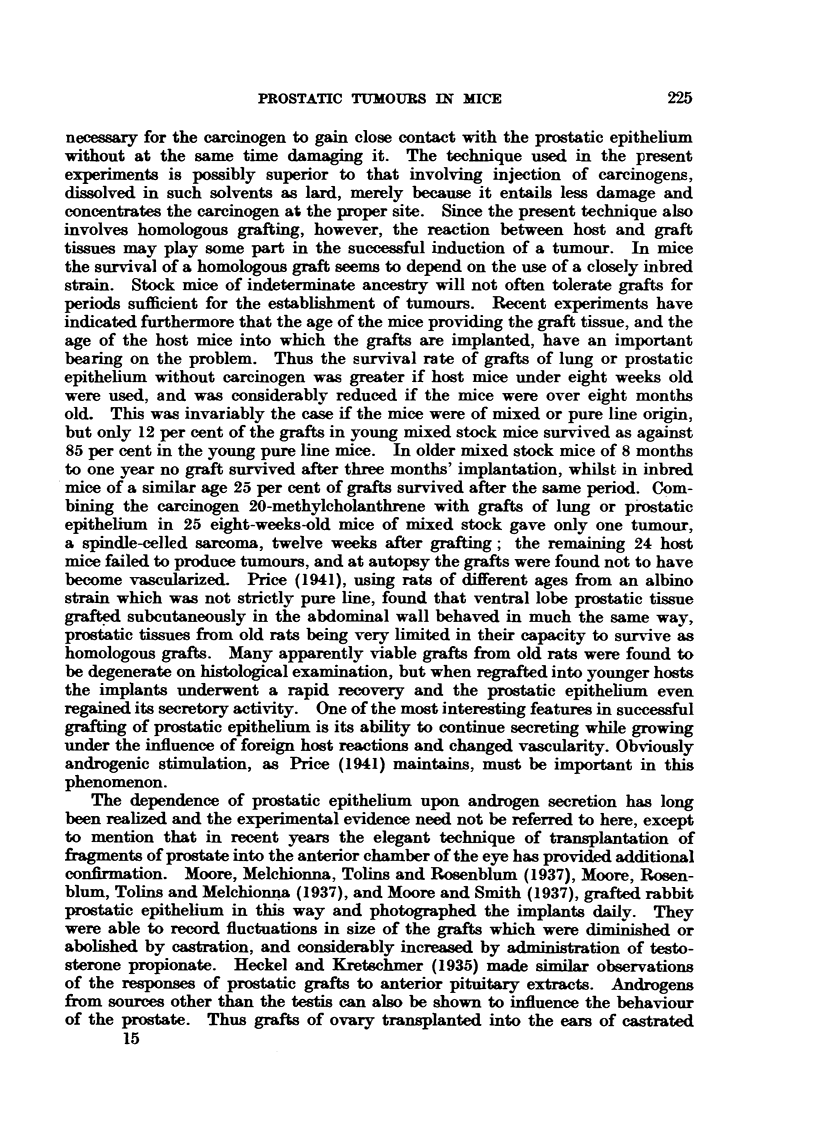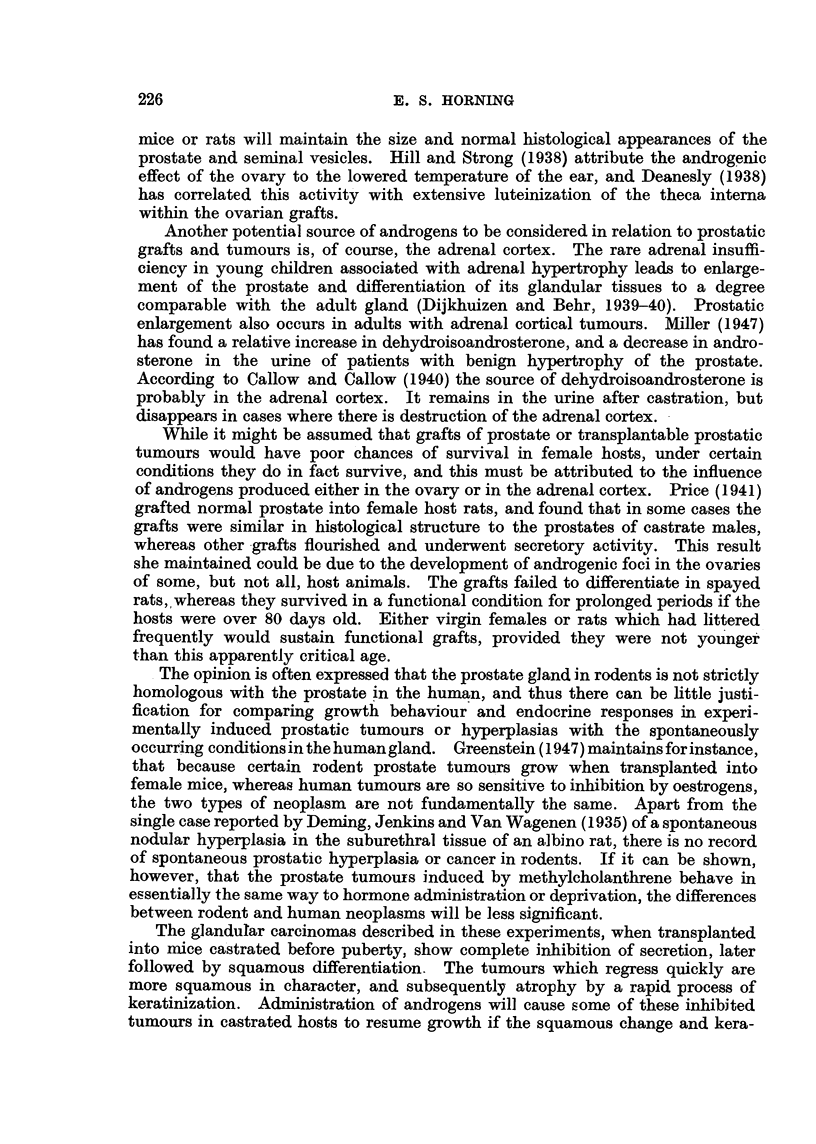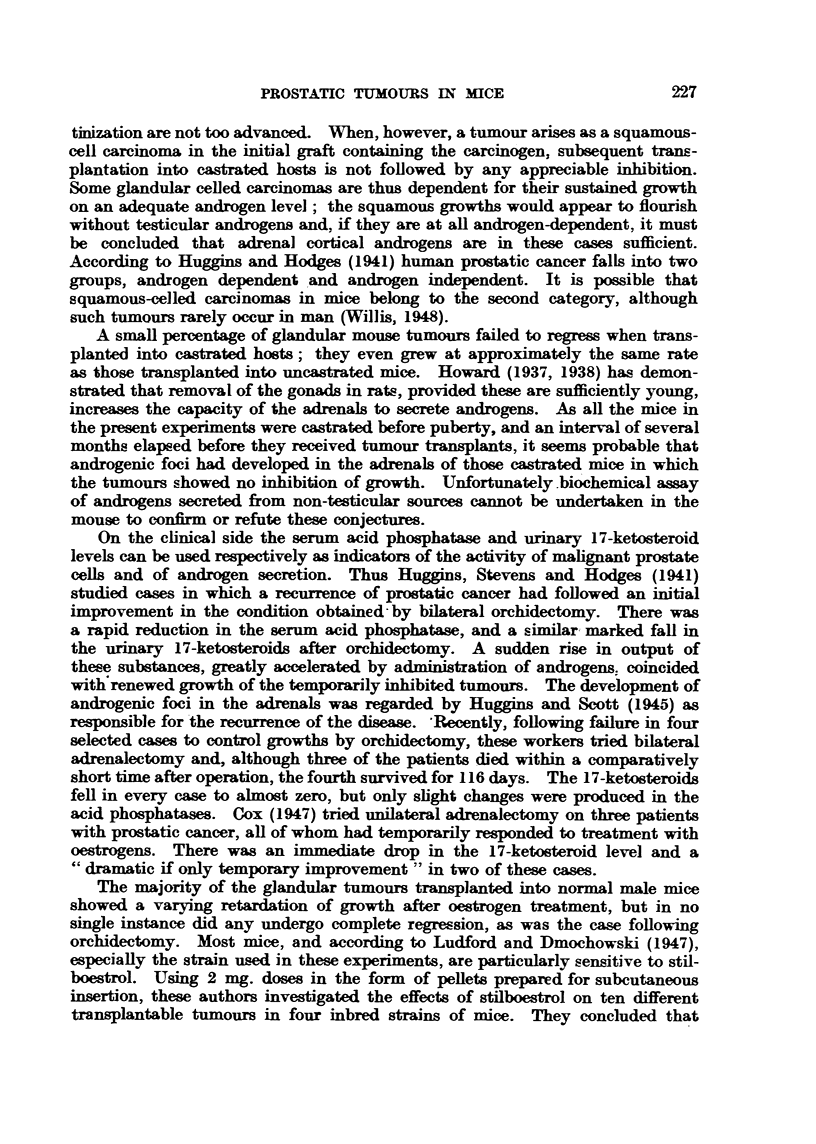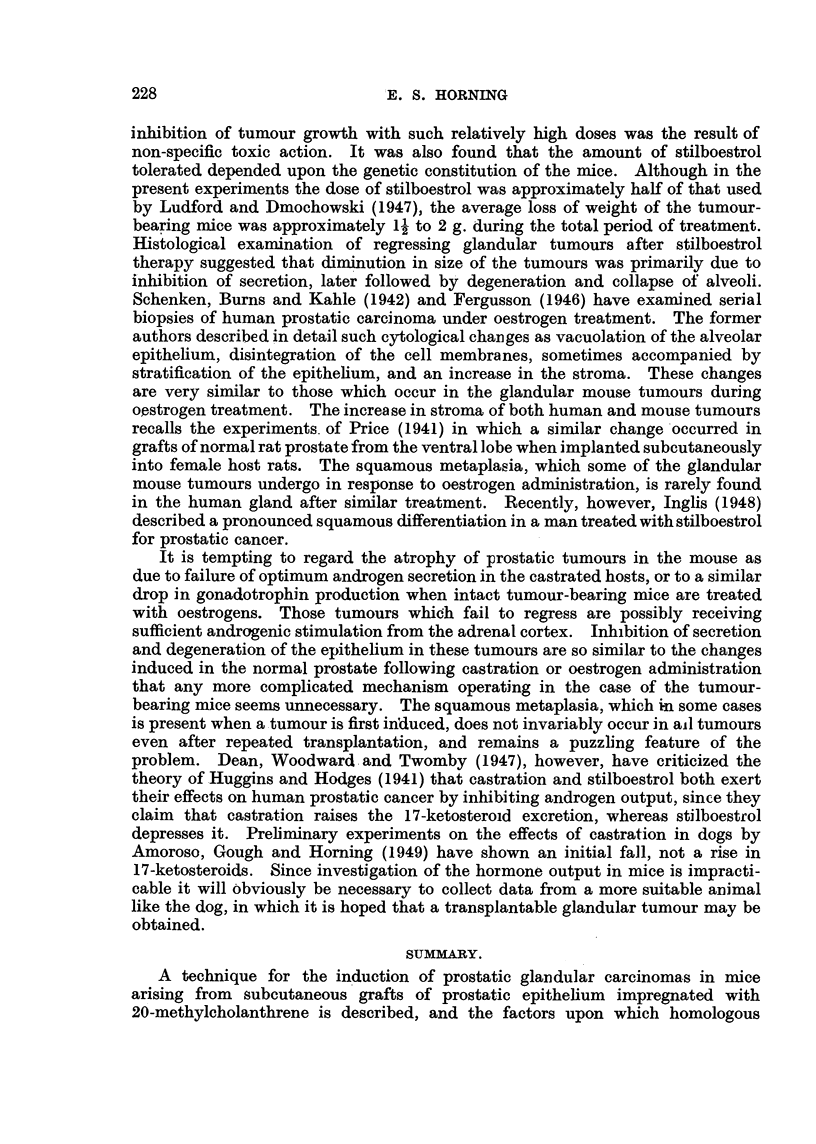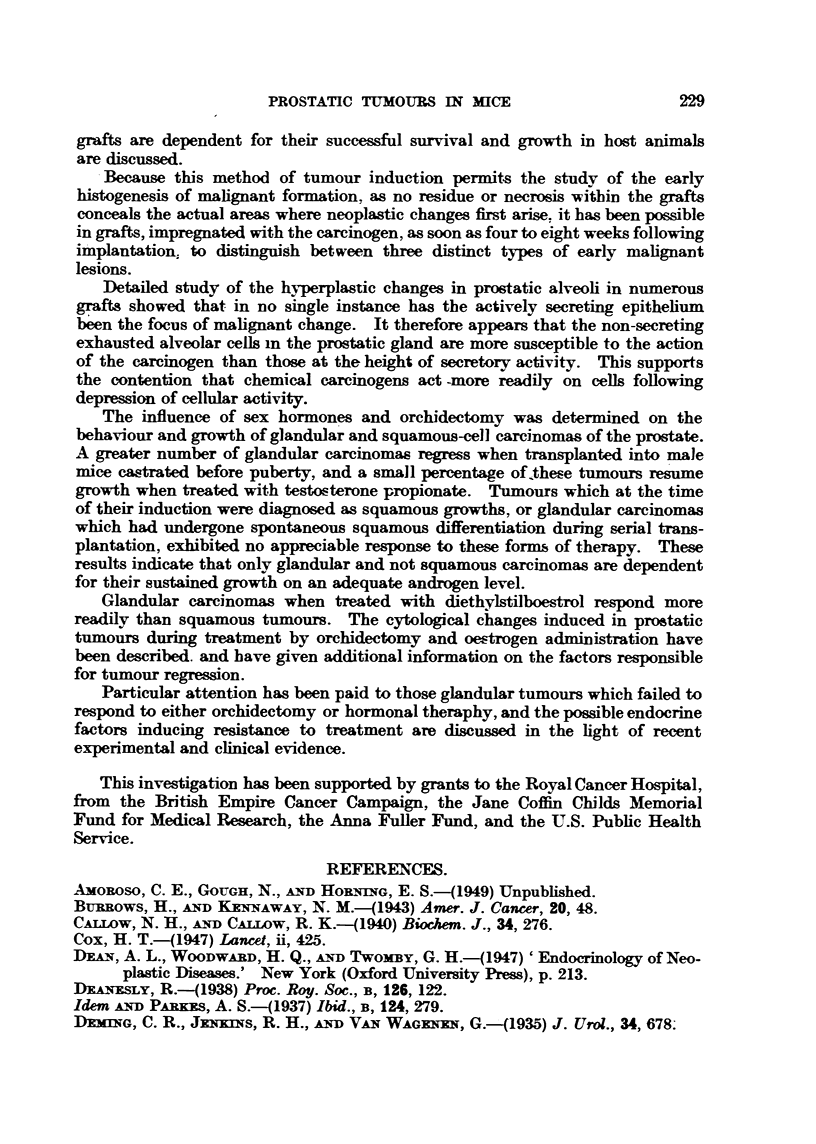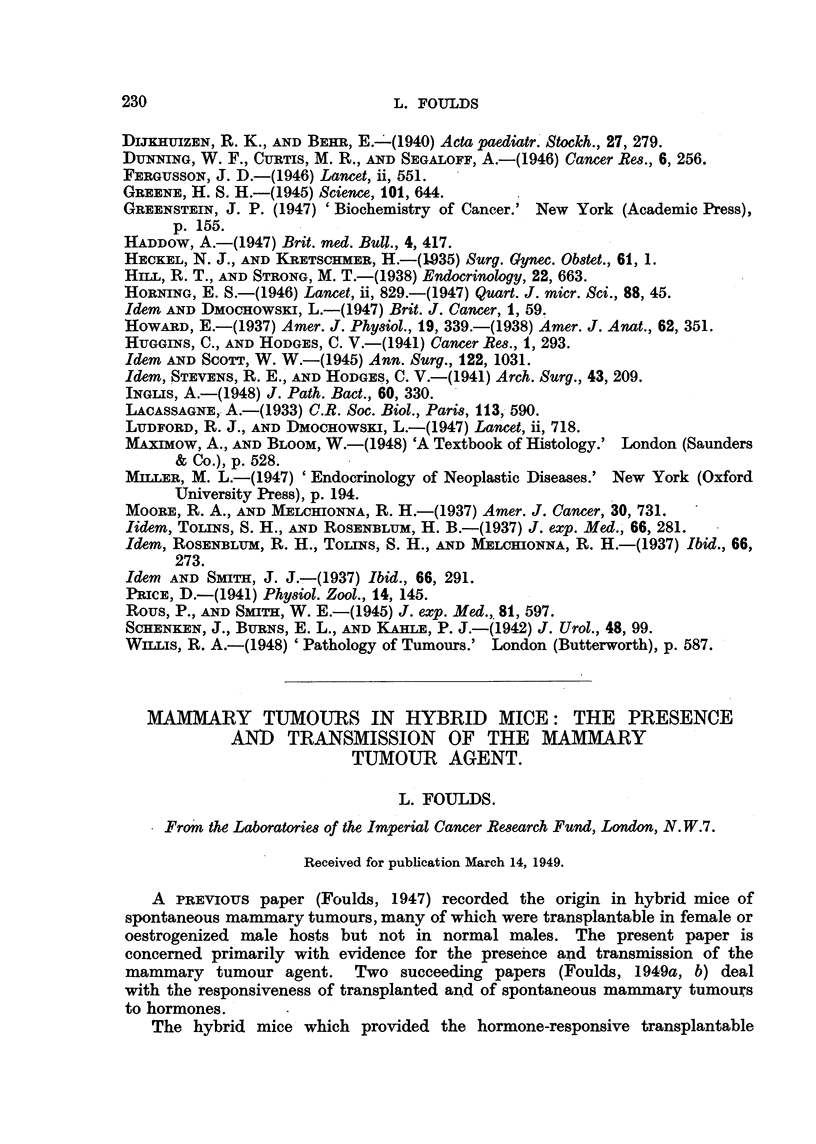# The Effects of Castration and Stilboestrol on Prostatic Tumours in Mice

**DOI:** 10.1038/bjc.1949.22

**Published:** 1949-06

**Authors:** E. S. Horning

## Abstract

**Images:**


### THE EFFECTS OF CASTRATION AND STILBOESTROL ON PROSTATIC TUMOURS IN MICE

E. S. HORNING.

*From The Chester Beatty Research Institute, Royal Cancer Hospital, London, S.W 3*

Received for publication April 20, 1949.

IT is generally agreed that prostatic cancer does not develop spontaneously in rodents. Transplantable tumours of the rodent prostate have been induced following inoculation of the gland *in situ* with carcinogens, but the resulting growths have been either sarcomas or squamous-cell carcinomas (Moore and Melchionna, 1937 ; Dunning, Curtis and Segaloff, 1946 ; Horning and Dmochowski, 1947). Such kinds of prostatic cancer rarely occur, however, in man (Willis, 1948).

In view of the need for an experimental investigation into the effects of orchidectomy and endocrine therapy on the behaviour and growths of prostate tumours, new methods of tumour induction were undertaken in pure line mice, with the hope of obtaining a transplantable adeno-carcinoma. The successful induction of glandular carcinomas in mice arising from subcutaneous implants of adult prostatic epithelium impregnated with crystals of 20-methylcholanthrene has already been described in a preliminary communication (Horning, 1946)

The object of this paper is to trace in some detail the histogenesis of the induced prostatic carcinomas and to describe the influence of castration and sex hormone administration on their growth.

### TECHNIQUE.

The method of tumour induction consists in isolating small strips of prostatic epithelium from either the anterior, ventral or dorsal lobes of the gland, and wrapping the pieces of epithelium around crystals of 20-methylcholanthrene prior to grafting the whole subcutaneously into host mice of the same sex, age and strain. The fragments of epithelium were isolated under a dissecting microscope with the aid of an iridectomy knife. The carcinogen was then placed in contact with the surface of the epithelium, and inserted into a Bashford transplanting needle, care being taken to shield the carcinogen from the connective tissues of the host animal. In some instances as many as three subcutaneous primary grafts were made on each side of the belly of a single host mouse to give a series of prostatic tumours growing under identical hormonal conditions. The technique is similar in some essentials to that previously employed by Greene (1945), and Rous and Smith (1945), except for the important difference that the present experiments involved tumour production from adult, not embryonic tissues.

The influence of bilateral orchidectomy upon the growth rates of glandular and squamous cell prostatic carcinomas was investigated in 6-months-old mice, which had previously been castrated before attaining puberty. The tumours used in these experiments were in their twelfth passage of serial transplantation. Prostatic tumours undergoing regression, following transplantation into orchidectomized mice, were treated with testosterone propionate, each mouse receiving a dose of 1 mg. dissolved in oil every 24 hours.

Two types of prostatic carcinomas, an actively secreting glandular cell tumour and non-secreting squamous-cell tumour, both in their eighth generation of serial transplantation, were selected for determining the influence of stilboestrol on the behaviour of tumour growth. Each mouse in these groups received a 1 mg. pellet of diethylstilboestrol by subcutaneous implantation into its right flank according to the technique of Deanesly and Parkes (1937). The hormone pellets were inserted at the time of tumour transplantation, and the same dosage was repeated every 14 days.

The growth rates of all transplanted tumours were recorded weekly on silhouette charts.

All material for histological examination was fixed either in Zenker-formol or else in alcoholic Bouin, and subsequently stained with haematoxylin and eosin.

### RESULTS.

### *Occurrence of Tumours.*

Repeated experiments on tumour induction by this technique have demonstrated that neoplasms develop more rapidly, and arise in greater numbers, when prostatic epithelium is obtained from young mice in preference to old ones, provided the implants are grafted into host mice of the same sex, age and strain.

For instance, out of 25 male mice, old breeders over 10 months old, all of which received prostatic grafts impregnated with the carcinogen, only six developed palpable tumours after four months, whilst in a group of 25 young virgin males, 8 weeks' old, 19 developed tumours within eight to twelve weeks. For this reason prostatic tissue for purposes of grafting was always obtained from mice under twelve weeks of age.

The observations described in this communication were made on a total of 75 Strong A male mice of the above age. Mice in groups of 10 received grafts at intervals covering a period of 18 months. Small primary palpable tumours appeared from seven to twelve weeks after grafting. Out of a total of 75 treated mice, 54 developed tumours. Histological examination showed that 42 of these were glandular cell carcinomas. 10 were of the squamous variety, and 2 were spindle-cell sarcomas. Sixteen homologous grafts failed to grow, and were found at autopsy not to have become vascularized.

In order to determine the early phases of carcinogenesis within the primary grafts a further 30 mice received prostatic implants, and were sacrificed at intervals ranging from two weeks to five months after implantation. A control group of 35 mice received grafts of prostatic epithelium without the carcinogen, and were killed at intervals ranging from two weeks to six months after implantation.

### *Histogenesis of Tumours.*

Before describing the early malignant changes which develop in prostatic grafts following impregnation with the carcinogen, it will be advisable at this stage to discuss briefly the probable homologies, and also the anatomical differences which exist between the human and rodent prostates.

The mouse gland, like that of the human, arises as a series of outgrowths from the embryonic urethra at its point of origin from the urinary bladder. The mouse gland consists of paired anterior, ventral and dorsal lobes, together with a small median gland, which may be absent or considerably modified in certain pure line mice. The human prostate, on the other hand, consists of two lateral lobes, which are practically fused together, with an anterior portion and an isthmus or middle lobe which, as in the rodent, surrounds the urethra. Unlike the human gland, the mouse prostate has no uterus masculinus or its equivalent, nor is it encapsulated. A difference exists also in the number of ducts which open independently into the urethra. Thus in the human gland there are as many as 32 ducts (Maximow and Bloom 1948), whilst in the mouse there are only six. A fibromuscular stroma consisting of dense connective tissue is common to both types of gland, but the mouse gland possesses a less elastic arrangement of smooth muscle fibres which in the human prostate surround the alveoli. The secretory epithelium is almost identical in its histology in the two species except for minor variations in the appearance of a basement membrane, which is better defined in the mouse. In both the human and rodent glands the secretory epithelium is thrown into folds which project into the glandular lumina, and though this folding varies considerably with the functional state of the gland, it is more pronounced in the rodent gland (Fig. 2 and 3). The cytoplasm of the glandular epithelium in both species contains numerous secretory granules, the majority of which are of lipids. Cytologically treated mouse material shows the secretion in both forms to consist of protein with fine lipoidal droplets in suspension. The alveolar concretions of the human prostate form characteristic structures, frequently becoming calcified, whereas the stored secretion of the mouse gland is usually evenly dispersed and lacks concretions. Hyperplasia of the prostate never occurs spontaneously in rodents, but a similar condition arises in mice following prolonged treatment with oestrogen, which induces a pronounced metaplasia of the prostatic epithelium in the dorsal and ventral lobes, involving, as in man, urethral obstruction, retention of urine and bilateral hydronephrosis (Fig. 1).

*Control grafts implanted without the carcinogen.*

Isolated fragments of prostatic epithelium without carcinogen continue to secrete prostatic fluid following successful implantation under the skin of host mice of the same sex and strain (Fig. 4). In such control grafts, fixed one and six months after implantation, distension of the alveoli occurs to a degree far in excess of what is typical of the normal gland *in situ*, and leads frequently to a condition of cystic dilatation (Fig. 4 and 2). The degree of the alveolar distension varies considerably with the age of the graft Fig. 4 shows a section through a control implant from the dorsal lobe, fixed after 12 weeks, in which all the alveoli are considerably distended. In cytological preparations there is some indication that the secretion is deficient in lipids in grafted alveoli as compared with those in the normal intact gland. In both control and experimental grafts there is evidence that the secretion is slowly dispersed and absorbed within the tissues of the host. It will be noted that the epithelium in control grafts is not hyperplastic, and hence it is important to distinguish as clearly as possible between growth in the grafts containing the carcinogen which is primarily due to a failure in the release of the secretion, since the ducts are no longer patent, and proliferation which might be due to the direct action of the carcinogen.

*Neoplastic changes in grafts implanted with the carcinogen.*

The carcinogen, which is placed in direct contact with the living tissue without a solvent such as oil or lard, induces very little foreign body reaction, necrosis or residue within the graft which might mask or conceal the actual areas where neoplastic changes first arise. Thus it is possible to study serial sections of the primary grafts, and to trace each invading clump of malignant cells back to the individual hyperplastic alveolus from which it has arisen. Fig. 5 shows a section through a whole graft of dorsal lobe epithelium fixed $4\frac{1}{2}$ weeks after implantation. There is very little foreign body reaction, and it will be noted how clearly the foci in which malignant changes first arise may be detected. Adjacent to alveoli lined with a low columnar epithelium and distended with secretion containing epithelial debris and polymorphs are collapsed alveoli in which the epithelium has entered an exhaustion phase. The tall columnar cells, which are characteristic of collapsed alveoli, are folded and free of secretion probably owing to absorption by the host tissues.

Examination of grafts of various ages after implantation has shown conclusively that neoplasms are derived only from the epithelium which has entered the exhaustion phase of the secretory cycle. It is in these alveoli that hyperplastic epithelial changes are first seen (Fig. 8 and 9), following a phase of mitosis, abnormal cell division and pycnosis (Fig. 8). The hyperplasia is prominent in grafts four to five weeks old, giving an appearance to the epithelium which is closely similar to that seen in the human prostate during benign enlargement (Fig. 6 and 7). In no single instance in any of the numerous grafts examined has the actively secreting epithelium lining distended alveoli been the focus of neoplastic change. Presumably the non-secreting exhausted alveolar cells are more susceptible to the action of the carcinogen (Fig. 6). The hyperplastic changes in the mouse prostate are invariably accompanied by a pronounced increase in the fibromuscular stroma, and there is lymphocytic infiltration which varies considerably in different grafts of the same age (Fig. 8). In grafts five to eight weeks after implantation the alveolar epithelium contains patches which are 10–20 cells in depth (Fig. 10), with cellular proliferation predominant in the basal layers.

At this early stage it is possible to distinguish three distinct types of epithelial proliferation (A, B and C), which are illustrated respectively in Fig. 11, 12 and 13.

*Type A.*—Gives rise to a clump of darkly staining cells in which outlines are indistinct and there is considerable variation in nuclear size and structure (Fig. 11).

*Type B.*—Consists of pale staining cells undergoing squamous metaplasia (Fig. 12).

These two types of invasive new growth arise from alveolar epithelium.

*Type C.*—This more uncommon variety, characterized by a stratified squamous metaplasia of the epithelium *in situ*, followed by diffuse marginal growth into the stroma, clearly arises from duct epithelium (Fig. 13).

By studying the cytology and mode of growth of these early invasive cells through later phases of development in older grafts, it was possible to predict the types of tumours which would subsequently have arisen. The tongue-like colony of early malignant cells of Type A was typical of those tumours which finally developed into secreting glandular carcinomas. In the example illustrated the proliferation arose from a single alveolus and was the only focus of malignant change in this particular graft. Later stages from other grafts showed the onset of secretory activity, and from the main focus of proliferation malignant glandular cells were seen to migrate independently into the stroma, and finally to give rise to atypical alveoli along the margins of the tumour. The newly differentiated alveoli contained irregular low columnar epithelium actively engaged in secretion. Fig. 14 shows three malignant alveoli in the peripheral area of an invading new growth of this type from an eight weeks' old graft. Several epithelial cells are in process of abnormal cell division. The lumina are filled with secretion and cell debris. Portions of this particular primary glandular carcinoma, transplanted into mice of the same age and sex as the host mouse, retained their histological characters for 16 generations of serial transplants before finally transforming into a squamous-celled tumour.

The Type B lesion arose in the various grafts examined from a single alveolus, and the example illustrated in Fig. 12 was again the only neoplastic focus within the entire graft. The proliferating cells are confined to a relatively restricted area of the alveolar wall; elsewhere the epithelium is normal in structure. The malignant cells contain small nuclei with numerous atypical mitotic figures. Apart from the different morphology and staining reactions of the proliferating cells, it will be noted that they take origin from an alveolus in which secretory activity has been inhibited. Adjacent alveoli not showing these proliferative changes are distended with secretion. In older grafts the Type B proliferation leads to the formation of pseudo-alveoli at the margins of the tumour and, although the glandular architecture of the epithelium is thus partly restored, there is no evidence of secretion. The cells acquire the characteristic intercellular fibrils of "prickle" cells, and keratinization ensues at the periphery of each alveolus. From study of many examples of this type of growth in grafts of various ages, it seems clear that the Type B proliferation leads to the formation of a squamous-cell carcinoma in which alveolar formation is preserved.

It is unnecessary to give a detailed account of the Type C proliferation (Fig. 15), except to point out that the squamous cell carcinoma derived from it grows rapidly and extensively in the grafts, and never gives rise to pseudo-alveoli. Instead the usual cell nests are formed and keratinization results in the formation of keratin pearls. It is interesting to note that the epithelium of the ductus deferens remains unaffected where it is included in the original grafted fragment, and this may be due to the thick circular muscle coat resisting the diffusion of the carcinogen (Fig. 15).

A minority of the grafts examined showed separate foci of Type A and Type C proliferation within the same graft. Fig. 16 shows an early secreting adeno-carcinoma together with a squamous-cell carcinoma arising in the wall of an adjacent duct. There is some evidence that where the two varieties of malignant change co-exist in a primary graft the subsequent tumour becomes a squamous-cell carcinoma, which rapidly infiltrates the adeno-carcinomatous areas until all trace of the glandular tumour is lost.

*Influence of Bilateral Orchidectomy on Tumour Growth and Subsequent Treatment with Testosterone Propionate.*

*Glandular carcinoma.*

An actively secreting glandular carcinoma, already in its 12th generation of serial transplants, exhibiting no evidence of squamous differentiation, was selected to determine the influence of orchidectomy on tumour growths. The castrated and the intact normal control mice all received subcutaneous transplants from

EXPLANATION OF PLATES.

FIG. 1.—Photograph of a male R III mouse after twelve months' treatment with oestrogen. Application of the sex hormone by painting the skin on the nape of the neck has induced a pronounced metaplasia of the prostatic epithelium in the dorsal and ventral lobes, causing urethral obstruction, retention of urine and bilateral hydronephrosis.

FIG. 2.—Section through the dorsal prostate of a normal Strong A mouse, showing the manner in which the glandular epithelium is thrown into folds. The secretion of the mouse prostate gland is evenly dispersed and lacks concretions. Compare with that of human gland seen in Fig. 3 × 64.

FIG. 3.—Normal human prostate gland. The secretory epithelium like that in the mouse gland is thrown into folds which project into the glandular lumina. Note alveolar concretions, and compare with that of mouse in Fig. 2. × 64.

FIG. 4.—Implant of portion of dorsal epithelium from a normal Strong A mouse without carcinogen after surviving subcutaneously in a host male mouse of same age and strain for 12 weeks. Note distension of alveoli with prostatic fluid which is far in excess of what is typical of the normal gland *in situ*, and frequently leads to cystic dilatation. Compare with Fig. 2. × 27.

FIG. 5.—Section through a whole graft of dorsal lobe epithelium impregnated with 20-methylcholanthrene fixed after 4½ weeks' subcutaneous implantation in a host mouse. There is very little foreign body reaction to the carcinogen. Note hyperplastic alveoli, adjacent to which are alveoli lined with low columnar epithelium and distended with secretion. × 20.

FIG. 6.—Hypertrophy of prostatic epithelium obtained from dorsal lobe of a Strong A mouse, impregnated with carcinogen and fixed after 4½ weeks' subcutaneous growth in a host mouse of same age, sex and strain. Also note increase in the fibro-muscular stroma. × 75.

FIG. 7.—Simple hypertrophy in a human prostate. Compare with Fig. 6. × 63.

FIG. 8.—Enlarged portion of prostatic epithelium from section illustrated in Fig. 6. As the result of carcinogenic action, several epithelial cells are seen in division, some showing abnormal mitosis and pycnosis. × 260.

FIG. 9.—Early hyperplastic changes in prostatic epithelium of a Strong A mouse, after eight weeks' subcutaneous implantation with carcinogen in a host mouse of same sex, age and strain. The glandular epithelium contains patches which are 10–20 cells in depth. × 64.

FIG. 10.—Same as Fig. 9, showing hyperplastic area of alveolar epithelium 10 to 20 cells in depth, with cellular proliferation predominant in the basal layers. × 160.

FIG. 11.—Type A lesion. Epithelial proliferation in a subcutaneous graft of dorsal prostatic epithelium impregnated with 20-methylcholanthrene fixed after eight weeks' growth in a host mouse. This early malignant tongue of cells indicated by arrows has arisen from the glandular epithelium of an adjacent alveolus, and has invaded the fibromuscular stroma of the graft. × 105.

FIG. 12.—Type B lesion arose from the epithelium of a single alveolus in a subcutaneous graft impregnated with carcinogen, and fixed after 8½ weeks' growth. The proliferating cells (indicated by arrows) arose from a relatively restricted area of the alveolar wall, and from an alveolus in which secretory activity had been inhibited. × 115.

FIG. 13.—Type C lesion (illustrated by arrow) characterized by a stratified squamous metaplasia of the epithelium *in situ*, which has arisen from duct epithelium. × 53.

FIG. 14.—Three newly differentiated alveoli, consisting of irregular low columnar epithelium engaged in secretion. These malignant alveoli are in the peripheral area of an invading new growth of an eight-weeks-old graft. × 445.

FIG. 15.—Eight-weeks-old graft of dorsal lobe, showing early malignant changes in many alveoli. Note the epithelium of the ductus deferens (indicated by arrow) remains unaffected by action of the carcinogen. × 80.

FIG. 16.—7½-weeks-old graft of dorsal epithelium, with two varieties of malignant change co-existing in primary graft. One arrow indicates a squamous-cell carcinoma arising in the wall of a duct, and two arrows indicate an early glandular carcinoma. × 75.

1

2

3

4

Horning.

5 6 7 8

9 10 11 12

Horning.

13 14

15 16

the same tumour. All glandular tumours growing in intact normal mice showed a remarkably constant growth rate, and there was no tendency to regress throughout 14 generations of routine serial transplantation. In fact, owing to the rapid growth of this particular prostatic carcinoma, the tumour-bearing host mice either died or had to be killed during the 6th to 8th week following transplantation. Examination of the silhouette charts demonstrates the even and typical progressive growth of this tumour in the control group (Fig. 17).

All transplants grew during the first week in the castrated mice, but in the second week there was a slight inhibition of growth in comparison with those in

FIG. 17.—Silhouette chart illustrating the influence of bilateral orchidectomy, and of subsequent treatment with testosterone propionate, on the growth of transplantable glandular cell carcinomas of the prostate in Strong A mice. The tumours recorded here were in their 12th transplant generation.

the control group. One tumour (Fig. 17, No. 1) had completely regressed. By the end of the third week all tumours, with the exception of two which were growing at approximately the same rate as those in the controls, were rapidly regressing. On palpation the tumours undergoing regression were hard and nodular ; the two which failed to respond to orchidectomy were soft, and similar to those growing in the control mice. At the end of the 5th week five tumours had completely regressed, and three had ceased to exhibit any signs of growth (Fig. 17, No. 1, 2, 3, 6 and 9).

In order to determine the influence of the male sex hormone on the behaviour of tumour growth, the three mice still bearing regressing tumours at the end of the 5th week received every 24 hours a subcutaneous inoculation of testosterone propionate. Two tumours failed to respond to this treatment (Fig. 17, No. 5 and 10), and the remaining tumour, after an interval of one week, began to grow

FIG. 18.—Silhouette chart showing the effects of castration on a transplantable squamous cell carcinoma of the prostate in its 12th transplant generation in Strong A mice. This tumour was originally a glandular cell carcinoma which had undergone a spontaneous metaplasia during the 10th generation of serial transplantation.

again at a rate typical of the control tumours (Fig. 17, No. 4). This particular tumour when transplanted into host mice of the same sex, age and strain was carried on in serial transplants for six generations.

The variable influence of orchidectomy on the behaviour and growth of this glandular carcinoma was further demonstrated in another series of experiments which were repeated at a later date. In this instance two groups of mice, 10 in each, likewise castrated before puberty, received transplants of the same tumour. The growth rate of three tumours in the first group of 10 mice was entirely unaffected by castration. Four tumours in the second group also grew at approximately the same rate as the transplants in the intact control mice. Of 13 tumours out of a total of 20 in the two groups which responded to orchidectomy, 4 completely regressed, and the remaining 9 tumours exhibited marked inhibition of growth. A total of 6 mice, 3 in each group, whose tumours were retarded by orchidectomy, received similar inoculations of testosterone. Four tumours responded to this treatment by showing an immediate increase in growth rate while the remainder were unaffected.

*Squamous-cell carcinoma.*

A glandular cell carcinoma which had undergone spontaneous metaplasia after its 10th generation of serial transplants in non-castrated normal male mice was chosen to determine the possible influence of orchidectomy on a glandular tumour which was undergoing squamous transformation. Microscopic examination of this particular tumour had shown that the metaplastic alveolar epithelium was columnar and much of it still retained secretory activity, the alveoli being distended with secretion and containing isolated epithelial cells undergoing atypical mitosis. A variable degree of keratinization associated with prickle cell transformation of the epithelium occurred in concentric nests of cells in the peripheral alveoli of the tumour.

Comparison of the growth rates of this particular tumour transplanted into intact control mice, and into castrated mice, showed that orchidectomy had very little influence on the growth of a glandular carcinoma if squamous metaplasia with keratinization had already commenced in the tumour at the beginning of the experiment (Fig. 18). The occasional slight inhibition of tumour growth in some orchidectomized mice was within the limits of possible normal variation.

The histological changes accompanying variations in growth and behaviours in castrated and non-castrated mice, together with the endocrine factors involved in this process, will be discussed in another section of this paper.

### *Influence of Stilboestrol Treatment on Growth.*

A glandular carcinoma in its fifth generation of serial transplants and a squamous cell carcinoma in the sixth generation were selected for experiments with stilboestrol, and all tumours were transplanted into the left flanks of host mice. Pellets containing 1 mg. of diethylstilboestrol were implanted subcutaneously into the right flanks, the same dose being repeated every 14 days. Microscopical diagnosis of the tumours prior to transplantation showed that the glandular carcinoma was a fairly well differentiated and actively secreting tumour. The second type of tumour, since its induction as a primary new growth, had maintained its structure as a squamous cell carcinoma during serial transplantation and was not originally a glandular tumour which had undergone squamous metaplasia. From its first appearance this tumour exhibited prickle cells, keratinization and a deficiency of stroma.

*Glandular cell carcinoma.*

The influence of stilboestrol on this particular tumour was determined for a period of six weeks on a total of 50 mice. There were five separate experiments, each comprising 10 treated mice, with equal numbers of untreated controls. Fig. 19 shows the effects of the oestrogen on tumour growth in a batch of 10

FIG. 19.—Silhouette chart illustrating the influence of stilboestrol treatment on a glandular carcinoma of the prostate in its 6th generation of serial transplantation. K. = killed. + = died.

mice, and is representative of the general effect obtained throughout. Of 50 treated mice 38 showed a slight arrest of tumour growth, 15 displayed a more pronounced retardation, whilst the remaining tumours still continued to grow at approximately the same rate as in the controls. In no single instance did any of the treated tumours undergo a complete regression during the six weeks' period of treatment. Although the first dosage of stilboestrol did not appear to impair the health of the mice, at the end of three weeks' treatment the average loss of

*Squamous cell carcinoma.*

Unlike the glandular cell carcinoma none of the squamous tumours showed any pronounced sensitivity to stilboestrol treatment (Fig. 20). The same numbers of mice were used of similar age and treated with stilboestrol for the same duration. There was a delay, in comparison with the response of the glandular tumours, of three weeks before any effect of the oestrogen could be detected. Then approximately three out of ten in each of the five groups of mice showed a slight arrest of tumour growth. As these squamous tumours are normally hard and firm, no apparent change could be detected by palpation after four weeks' administration of oestrogen. By the sixth week the loss of body weight amounted to 1 g., and some mice had already died during the previous week.

### *Histological Changes Induced by Castration and by Stilboestrol Treatment.*

*Castration.*

*Glandular carcinomas.*—The tumours examined in greater detail have been selected according to their response to castration alone, or to castration followed by administration of testosterone propionate. Cytological examination was made eight weeks after transplantation.

Tumours exhibiting no inhibition of growth following castration, e.g. No. 7 in Fig. 17, were harder and more nodular on palpation than those growing in control intact mice. Secretory activity as judged from sections had been suppressed and squamous metaplasia, of variable intensity in different areas of any tumour, was always found. The prostatic alveoli still retained their characteristic form and, although secretion appeared to be inhibited, many lumina contained prostatic coagulum and cell debris. Keratinization was restricted to cells at the periphery of the alveoli (Fig. 21). In Tumour 8, Fig. 17, there was no keratinization, but many epithelial cells possessed intercellular fibrils.

Tumours showing considerable regression of growth in response to castration underwent more pronounced cytological changes. Thus in the extreme case of Tumour 5, Fig. 17, which had completely regressed and had shown no response to testosterone, islands of degenerating epithelium lying in a stroma which was largely replaced by keratin formed the characteristic histological picture. Epithelial degeneration was almost complete (see Fig. 22), and the interior of the

---

EXPANATION OF PLATES.

FIG. 21.—Tumour 7 in Fig. 17, showing suppression of secretory activity, squamous metaplasia and keratinization of cells at periphery of alveoli in a glandular carcinoma following transplantation into a male mouse castrated before puberty. Although this tumour exhibited no inhibition of growth, it underwent pronounced histological changes during transplantation. × 390.

FIG. 22.—Tumour 5 in Fig. 17, illustrating a tumour which had completely regressed following transplantation into a castrated mouse, and failed to resume growth following treatment with testosterone propionate. Epithelial degeneration is complete, and interior of tumour consists largely of keratin. × 390.

FIG. 23.—Tumour 4, Fig. 17. The alveolar character of the glandular carcinoma persists in this tumour, which has undergone squamous metaplasia after transplantation into an orchidectomized mouse. This tumour regressed in response to castration, and resumed growth following treatment of host mouse with testosterone proprionate. × 390.

FIG. 24.—Alveolus of a glandular carcinoma eight days after treatment of host mouse with stilboestrol. There is a complete inhibition of secretory activity of glandular epithelium, and a slight shrinkage of nuclei. × 450.

FIG. 25.—Alveolus of a glandular tumour 3½ weeks after commencement of treatment of host mouse with stilboestrol. Note the vacuolation (indicated by arrows) of secretory epithelium, which is largely confined to the basal region of epithelial cells. Several nuclei have undergone chromatolysis. There is also a complete inhibition of secretory activity. × 450.

FIG. 26.—Alveolus of a tumour which responded to treatment five weeks following administration of stilboestrol. There is a marked reduction in height of epithelium, and most nuclei are pycnotic. The vacuolation has resulted in rupture of apical cell membrane, with expulsion of cell contents into the lumina of the alveoli. × 340.

21

22

23

24

25

26

tumour consisted largely of keratin. There was, of course, no mitotic activity and in fact the cells were shrunken to scanty fringes of cytoplasm containing nuclei in advanced stages of chromatolysis. Portions of this tumour failed to grow when transplanted into normal host mice.

Tumours inhibited by castration but subsequently treated with testosterone, after which growth was renewed, were examined at the end of the third week of hormone administration, e.g. Tumour 4, Fig. 17. A similar squamous metaplasia with inhibition of secretion was found together with considerable mitotic activity (Fig. 23). The alveolar character of the growth had persisted, but the epithelial cells were shorter than in the original untreated tumour. These results were confirmed in a second series of experiments involving the same number of castrated mice using the same dosages of testosterone. There was abundant mitotic activity in these tumours responding to testosterone, and these grew successfully for six generations when transplanted into normal male mice. The squamous character of the tumour was retained with a uniform growth rate until the conclusion of the experiment. The tumours which failed to respond to testosterone had undergone extreme keratinization, and the epithelial islands consisted as before of degenerating cells (Fig. 22).

*Squamous cell carcinomas.*

Tumours which from the time of their induction had been diagnosed as squamous cell carcinomas were similarly transplanted into mice castrated before puberty. Examination of Fig. 18 shows that castration had no appreciable influence on squamous tumours of this type. There were no specific cytological changes except in those tumours (No. 2, 5, 9 and 10, Fig. 18) in which a slight retardation of growth coincided with patches of keratinization leading to the formation of keratin pearls.

*Stilboestrol treatment.*

*Glandular carcinoma.*—Unfortunately stilboestrol has a toxic effect on this special strain of mice, and tumour regression was in nearly every case accompanied by loss in weight of each tumour-bearing mouse (Fig. 19). A dosage of 1 mg. did not appear to impair the health of the mice until the end of the second week's treatment, after which three deaths occurred. The average loss of weight per mouse at the end of two weeks' treatment was approximately 2·5 g.

Complete inhibition of secretory activity of the prostatic epithelium occurred eight days after treatment, and following this the oestrogen appeared to induce two distinct kinds of histological change in the alveolar epithelium of this particular prostatic carcinoma: (*a*) a squamous metaplasia similar to that which has been described by several authors (Lacassagne, 1933; Burrows and Kennaway, 1934; Horning, 1947) for the prostate glands of normal intact rodents, or (*b*) a progressive vacuolation of the glandular cells, finally leading to disintegration of the alveoli. This latter type of response to treatment, though more uncommon in the rodent, is similar to that reported in man following stilboestrol treatment for prostatic carcinoma (Schenken, Burns and Kahle, 1942), and will be described in detail.

The cytological changes after varying periods of treatment all follow a similar pattern, although the latent period before onset varies slightly in individual mice. In the majority of mice, after three to four weeks' treatment, a vacuolation occurs in the basal cytoplasm of the glandular cells, with or without stratification of the epithelium. This process is more pronounced in those tumours which show a more marked inhibition of growth. Tumours found to be retarded in growth during the fifth week of treatment were characterized by a decrease in the size of the majority of the alveoli, a reduction in the height of the epithelium, a complete disappearance of cell-division, and an increase in the fibro-muscular stroma. The nuclei had become smaller, and many were hyperchromatic and later pycnotic. The most conspicuous cytoplasmic change seen in Bouin-fixed material was the vacuolation restricted at first to the basal region of the cells (Figs. 24 and 25). In many alveoli these vacuoles varied considerably in size, and often displaced the pycnotic nuclei towards the apical end of the distorted cells. Tumour material fixed in osmium tetroxide five weeks after oestrogen treatment showed the vacuoles to contain lipid droplets. The alveoli lined by stratified epithelium of varying thickness in different tumours did not possess any appreciable epithelial vacuolation.

In most regressing tumours intensified vacuolation of the glandular cells finally resulted in the rupture of the apical cell membrane, with expulsion of the cell contents into the lumina of the alveoli (Fig. 26). The alveoli diminished in size when this rupture occurred, whereas adjacent alveoli with intact epithelial cells appeared to be so slightly affected by the oestrogen that little more than a reduction in the height of the cells could be observed. Tumours after five to seven weeks' treatment grew erratically when first transplanted into normal mice during the first three generations of serial transplants. In subsequent generations these tumours invariably assumed a constant and progressive rate of growth, as though they had overcome what was only a temporary inhibition in response to stilboestrol.

### DISCUSSION.

The technique adopted in these experiments of inducing tumours of the prostate gland in mice lends itself admirably to the study of the early histogenesis of malignant formation, partly because the sites where neoplastic changes first occur are uncomplicated by foreign body reaction or necrosis within the graft. It was possible in grafts impregnated with carcinogens to follow in four to six weeks after implantation hyperplastic changes in the glandular epithelium, in which three distinct types of early malignancy could be distinguished. The types of tumours which would have developed subsequently could be predicted, even though cellular proliferations had occurred in many instances from only a single alveolus. A detailed study of the hyperplastic changes in numerous grafts has shown conclusively that in no single instance has an actively secreting epithelium been the focus of malignant change. The non-secreting exhausted alveolar cells appear to be more susceptible to the action of the carcinogen than those at the height of secretory activity. This observation supports Haddow's (1947) contention that chemical carcinogens act more readily on cells following depression of their functional activity.

It is difficult to interpret the reasons why previous attempts (Dunning, Curtis and Segaloff, 1946 ; Horning and Dmochowski, 1947) to obtain glandular carcinomas by injecting the prostate glands of living rodents *in situ* with carcinogens have only succeeded in inducing squamous or sarcomatous growths. Obviously there are several factors involved, and one of them might be that it is necessary for the carcinogen to gain close contact with the prostatic epithelium without at the same time damaging it. The technique used in the present experiments is possibly superior to that involving injection of carcinogens, dissolved in such solvents as lard, merely because it entails less damage and concentrates the carcinogen at the proper site. Since the present technique also involves homologous grafting, however, the reaction between host and graft tissues may play some part in the successful induction of a tumour. In mice the survival of a homologous graft seems to depend on the use of a closely inbred strain. Stock mice of indeterminate ancestry will not often tolerate grafts for periods sufficient for the establishment of tumours. Recent experiments have indicated furthermore that the age of the mice providing the graft tissue, and the age of the host mice into which the grafts are implanted, have an important bearing on the problem. Thus the survival rate of grafts of lung or prostatic epithelium without carcinogen was greater if host mice under eight weeks old were used, and was considerably reduced if the mice were over eight months old. This was invariably the case if the mice were of mixed or pure line origin, but only 12 per cent of the grafts in young mixed stock mice survived as against 85 per cent in the young pure line mice. In older mixed stock mice of 8 months to one year no graft survived after three months' implantation, whilst in inbred mice of a similar age 25 per cent of grafts survived after the same period. Combining the carcinogen 20-methylcholanthrene with grafts of lung or prostatic epithelium in 25 eight-weeks-old mice of mixed stock gave only one tumour, a spindle-celled sarcoma, twelve weeks after grafting; the remaining 24 host mice failed to produce tumours, and at autopsy the grafts were found not to have become vascularized. Price (1941), using rats of different ages from an albino strain which was not strictly pure line, found that ventral lobe prostatic tissue grafted subcutaneously in the abdominal wall behaved in much the same way, prostatic tissues from old rats being very limited in their capacity to survive as homologous grafts. Many apparently viable grafts from old rats were found to be degenerate on histological examination, but when regrafted into younger hosts the implants underwent a rapid recovery and the prostatic epithelium even regained its secretory activity. One of the most interesting features in successful grafting of prostatic epithelium is its ability to continue secreting while growing under the influence of foreign host reactions and changed vascularity. Obviously androgenic stimulation, as Price (1941) maintains, must be important in this phenomenon.

The dependence of prostatic epithelium upon androgen secretion has long been realized and the experimental evidence need not be referred to here, except to mention that in recent years the elegant technique of transplantation of fragments of prostate into the anterior chamber of the eye has provided additional confirmation. Moore, Melchionna, Tolins and Rosenblum (1937), Moore, Rosenblum, Tolins and Melchionna (1937), and Moore and Smith (1937), grafted rabbit prostatic epithelium in this way and photographed the implants daily. They were able to record fluctuations in size of the grafts which were diminished or abolished by castration, and considerably increased by administration of testosterone propionate. Heckel and Kretschmer (1935) made similar observations of the responses of prostatic grafts to anterior pituitary extracts. Androgens from sources other than the testis can also be shown to influence the behaviour of the prostate. Thus grafts of ovary transplanted into the ears of castrated mice or rats will maintain the size and normal histological appearances of the prostate and seminal vesicles. Hill and Strong (1938) attribute the androgenic effect of the ovary to the lowered temperature of the ear, and Deanesly (1938) has correlated this activity with extensive luteinization of the theca interna within the ovarian grafts.

Another potential source of androgens to be considered in relation to prostatic grafts and tumours is, of course, the adrenal cortex. The rare adrenal insufficiency in young children associated with adrenal hypertrophy leads to enlargement of the prostate and differentiation of its glandular tissues to a degree comparable with the adult gland (Dijkhuizen and Behr, 1939–40). Prostatic enlargement also occurs in adults with adrenal cortical tumours. Miller (1947) has found a relative increase in dehydroisoandrosterone, and a decrease in androsterone in the urine of patients with benign hypertrophy of the prostate. According to Callow and Callow (1940) the source of dehydroisoandrosterone is probably in the adrenal cortex. It remains in the urine after castration, but disappears in cases where there is destruction of the adrenal cortex.

While it might be assumed that grafts of prostate or transplantable prostatic tumours would have poor chances of survival in female hosts, under certain conditions they do in fact survive, and this must be attributed to the influence of androgens produced either in the ovary or in the adrenal cortex. Price (1941) grafted normal prostate into female host rats, and found that in some cases the grafts were similar in histological structure to the prostates of castrate males, whereas other grafts flourished and underwent secretory activity. This result she maintained could be due to the development of androgenic foci in the ovaries of some, but not all, host animals. The grafts failed to differentiate in spayed rats, whereas they survived in a functional condition for prolonged periods if the hosts were over 80 days old. Either virgin females or rats which had littered frequently would sustain functional grafts, provided they were not younger than this apparently critical age.

The opinion is often expressed that the prostate gland in rodents is not strictly homologous with the prostate in the human, and thus there can be little justification for comparing growth behaviour and endocrine responses in experimentally induced prostatic tumours or hyperplasias with the spontaneously occurring conditions in the human gland. Greenstein (1947) maintains for instance, that because certain rodent prostate tumours grow when transplanted into female mice, whereas human tumours are so sensitive to inhibition by oestrogens, the two types of neoplasm are not fundamentally the same. Apart from the single case reported by Deming, Jenkins and Van Wagenen (1935) of a spontaneous nodular hyperplasia in the suburethral tissue of an albino rat, there is no record of spontaneous prostatic hyperplasia or cancer in rodents. If it can be shown, however, that the prostate tumours induced by methylcholanthrene behave in essentially the same way to hormone administration or deprivation, the differences between rodent and human neoplasms will be less significant.

The glandular carcinomas described in these experiments, when transplanted into mice castrated before puberty, show complete inhibition of secretion, later followed by squamous differentiation. The tumours which regress quickly are more squamous in character, and subsequently atrophy by a rapid process of keratinization. Administration of androgens will cause some of these inhibited tumours in castrated hosts to resume growth if the squamous change and keratinization are not too advanced. When, however, a tumour arises as a squamous-cell carcinoma in the initial graft containing the carcinogen, subsequent transplantation into castrated hosts is not followed by any appreciable inhibition. Some glandular celled carcinomas are thus dependent for their sustained growth on an adequate androgen level; the squamous growths would appear to flourish without testicular androgens and, if they are at all androgen-dependent, it must be concluded that adrenal cortical androgens are in these cases sufficient. According to Huggins and Hodges (1941) human prostatic cancer falls into two groups, androgen dependent and androgen independent. It is possible that squamous-celled carcinomas in mice belong to the second category, although such tumours rarely occur in man (Willis, 1948).

A small percentage of glandular mouse tumours failed to regress when transplanted into castrated hosts; they even grew at approximately the same rate as those transplanted into uncastrated mice. Howard (1937, 1938) has demonstrated that removal of the gonads in rats, provided these are sufficiently young, increases the capacity of the adrenals to secrete androgens. As all the mice in the present experiments were castrated before puberty, and an interval of several months elapsed before they received tumour transplants, it seems probable that androgenic foci had developed in the adrenals of those castrated mice in which the tumours showed no inhibition of growth. Unfortunately biochemical assay of androgens secreted from non-testicular sources cannot be undertaken in the mouse to confirm or refute these conjectures.

On the clinical side the serum acid phosphatase and urinary 17-ketosteroid levels can be used respectively as indicators of the activity of malignant prostate cells and of androgen secretion. Thus Huggins, Stevens and Hodges (1941) studied cases in which a recurrence of prostatic cancer had followed an initial improvement in the condition obtained by bilateral orchidectomy. There was a rapid reduction in the serum acid phosphatase, and a similar marked fall in the urinary 17-ketosteroids after orchidectomy. A sudden rise in output of these substances, greatly accelerated by administration of androgens, coincided with renewed growth of the temporarily inhibited tumours. The development of androgenic foci in the adrenals was regarded by Huggins and Scott (1945) as responsible for the recurrence of the disease. Recently, following failure in four selected cases to control growths by orchidectomy, these workers tried bilateral adrenalectomy and, although three of the patients died within a comparatively short time after operation, the fourth survived for 116 days. The 17-ketosteroids fell in every case to almost zero, but only slight changes were produced in the acid phosphatases. Cox (1947) tried unilateral adrenalectomy on three patients with prostatic cancer, all of whom had temporarily responded to treatment with oestrogens. There was an immediate drop in the 17-ketosteroid level and a "dramatic if only temporary improvement" in two of these cases.

The majority of the glandular tumours transplanted into normal male mice showed a varying retardation of growth after oestrogen treatment, but in no single instance did any undergo complete regression, as was the case following orchidectomy. Most mice, and according to Ludford and Dmochowski (1947), especially the strain used in these experiments, are particularly sensitive to stilboestrol. Using 2 mg. doses in the form of pellets prepared for subcutaneous insertion, these authors investigated the effects of stilboestrol on ten different transplantable tumours in four inbred strains of mice. They concluded that inhibition of tumour growth with such relatively high doses was the result of non-specific toxic action. It was also found that the amount of stilboestrol tolerated depended upon the genetic constitution of the mice. Although in the present experiments the dose of stilboestrol was approximately half of that used by Ludford and Dmochowski (1947), the average loss of weight of the tumour-bearing mice was approximately $1\frac{1}{2}$ to 2 g. during the total period of treatment. Histological examination of regressing glandular tumours after stilboestrol therapy suggested that diminution in size of the tumours was primarily due to inhibition of secretion, later followed by degeneration and collapse of alveoli. Schenken, Burns and Kahle (1942) and Fergusson (1946) have examined serial biopsies of human prostatic carcinoma under oestrogen treatment. The former authors described in detail such cytological changes as vacuolation of the alveolar epithelium, disintegration of the cell membranes, sometimes accompanied by stratification of the epithelium, and an increase in the stroma. These changes are very similar to those which occur in the glandular mouse tumours during oestrogen treatment. The increase in stroma of both human and mouse tumours recalls the experiments of Price (1941) in which a similar change occurred in grafts of normal rat prostate from the ventral lobe when implanted subcutaneously into female host rats. The squamous metaplasia, which some of the glandular mouse tumours undergo in response to oestrogen administration, is rarely found in the human gland after similar treatment. Recently, however, Inglis (1948) described a pronounced squamous differentiation in a man treated with stilboestrol for prostatic cancer.

It is tempting to regard the atrophy of prostatic tumours in the mouse as due to failure of optimum androgen secretion in the castrated hosts, or to a similar drop in gonadotrophin production when intact tumour-bearing mice are treated with oestrogens. Those tumours which fail to regress are possibly receiving sufficient androgenic stimulation from the adrenal cortex. Inhibition of secretion and degeneration of the epithelium in these tumours are so similar to the changes induced in the normal prostate following castration or oestrogen administration that any more complicated mechanism operating in the case of the tumour-bearing mice seems unnecessary. The squamous metaplasia, which in some cases is present when a tumour is first induced, does not invariably occur in all tumours even after repeated transplantation, and remains a puzzling feature of the problem. Dean, Woodward and Twomby (1947), however, have criticized the theory of Huggins and Hodges (1941) that castration and stilboestrol both exert their effects on human prostatic cancer by inhibiting androgen output, since they claim that castration raises the 17-ketosteroid excretion, whereas stilboestrol depresses it. Preliminary experiments on the effects of castration in dogs by Amoroso, Gough and Horning (1949) have shown an initial fall, not a rise in 17-ketosteroids. Since investigation of the hormone output in mice is impracticable it will obviously be necessary to collect data from a more suitable animal like the dog, in which it is hoped that a transplantable glandular tumour may be obtained.

### SUMMARY.

A technique for the induction of prostatic glandular carcinomas in mice arising from subcutaneous grafts of prostatic epithelium impregnated with 20-methylcholanthrene is described, and the factors upon which homologous grafts are dependent for their successful survival and growth in host animals are discussed.

Because this method of tumour induction permits the study of the early histogenesis of malignant formation, as no residue or necrosis within the grafts conceals the actual areas where neoplastic changes first arise, it has been possible in grafts, impregnated with the carcinogen, as soon as four to eight weeks following implantation, to distinguish between three distinct types of early malignant lesions.

Detailed study of the hyperplastic changes in prostatic alveoli in numerous grafts showed that in no single instance has the actively secreting epithelium been the focus of malignant change. It therefore appears that the non-secreting exhausted alveolar cells in the prostatic gland are more susceptible to the action of the carcinogen than those at the height of secretory activity. This supports the contention that chemical carcinogens act more readily on cells following depression of cellular activity.

The influence of sex hormones and orchidectomy was determined on the behaviour and growth of glandular and squamous-cell carcinomas of the prostate. A greater number of glandular carcinomas regress when transplanted into male mice castrated before puberty, and a small percentage of these tumours resume growth when treated with testosterone propionate. Tumours which at the time of their induction were diagnosed as squamous growths, or glandular carcinomas which had undergone spontaneous squamous differentiation during serial transplantation, exhibited no appreciable response to these forms of therapy. These results indicate that only glandular and not squamous carcinomas are dependent for their sustained growth on an adequate androgen level.

Glandular carcinomas when treated with diethylstilboestrol respond more readily than squamous tumours. The cytological changes induced in prostatic tumours during treatment by orchidectomy and oestrogen administration have been described, and have given additional information on the factors responsible for tumour regression.

Particular attention has been paid to those glandular tumours which failed to respond to either orchidectomy or hormonal theraphy, and the possible endocrine factors inducing resistance to treatment are discussed in the light of recent experimental and clinical evidence.

This investigation has been supported by grants to the Royal Cancer Hospital, from the British Empire Cancer Campaign, the Jane Coffin Childs Memorial Fund for Medical Research, the Anna Fuller Fund, and the U.S. Public Health Service.

### REFERENCES.

AMOROSO, C. E., GOUGH, N., AND HORNING, E. S.—(1949) Unpublished.

BURROWS, H., AND KENNAWAY, N. M.—(1943) *Amer. J. Cancer*, **20**, 48.

CALLOW, N. H., AND CALLOW, R. K.—(1940) *Biochem. J.*, **34**, 276.

COX, H. T.—(1947) *Lancet*, ii, 425.

DEAN, A. L., WOODWARD, H. Q., AND TWOMBY, G. H.—(1947) 'Endocrinology of Neoplastic Diseases.' New York (Oxford University Press), p. 213.

DEANESLY, R.—(1938) *Proc. Roy. Soc.*, B, **126**, 122.

*Idem* AND PARKES, A. S.—(1937) *Ibid.*, B, **124**, 279.

DEMING, C. R., JENKINS, R. H., AND VAN WAGENEN, G.—(1935) *J. Urol.*, **34**, 678.

DIJKHUIZEN, R. K., AND BEHR, E.—(1940) *Acta paediatr. Stockh.*, **27**, 279.
DUNNING, W. F., CURTIS, M. R., AND SEGALOFF, A.—(1946) *Cancer Res.*, **6**, 256.
FERGUSSON, J. D.—(1946) *Lancet*, ii, 551.
GREENE, H. S. H.—(1945) *Science*, **101**, 644.
GREENSTEIN, J. P. (1947) 'Biochemistry of Cancer.' New York (Academic Press), p. 155.
HADDOW, A.—(1947) *Brit. med. Bull.*, **4**, 417.
HECKEL, N. J., AND KRETSCHMER, H.—(1935) *Surg. Gynec. Obstet.*, **61**, 1.
HILL, R. T., AND STRONG, M. T.—(1938) *Endocrinology*, **22**, 663.
HORNING, E. S.—(1946) *Lancet*, ii, 829.—(1947) *Quart. J. micr. Sci.*, **88**, 45.
*Idem* AND DMOCHOWSKI, L.—(1947) *Brit. J. Cancer*, **1**, 59.
HOWARD, E.—(1937) *Amer. J. Physiol.*, **19**, 339.—(1938) *Amer. J. Anat.*, **62**, 351.
HUGGINS, C., AND HODGES, C. V.—(1941) *Cancer Res.*, **1**, 293.
*Idem* AND SCOTT, W. W.—(1945) *Ann. Surg.*, **122**, 1031.
*Idem*, STEVENS, R. E., AND HODGES, C. V.—(1941) *Arch. Surg.*, **43**, 209.
INGLIS, A.—(1948) *J. Path. Bact.*, **60**, 330.
LACASSAGNE, A.—(1933) *C.R. Soc. Biol., Paris*, **113**, 590.
LUDFORD, R. J., AND DMOCHOWSKI, L.—(1947) *Lancet*, ii, 718.
MAXIMOW, A., AND BLOOM, W.—(1948) 'A Textbook of Histology.' London (Saunders & Co.), p. 528.
MILLER, M. L.—(1947) 'Endocrinology of Neoplastic Diseases.' New York (Oxford University Press), p. 194.
MOORE, R. A., AND MELCHIONNA, R. H.—(1937) *Amer. J. Cancer*, **30**, 731.
*Iidem*, TOLINS, S. H., AND ROSENBLUM, H. B.—(1937) *J. exp. Med.*, **66**, 281.
*Idem*, ROSENBLUM, R. H., TOLINS, S. H., AND MELCHIONNA, R. H.—(1937) *Ibid.*, **66**, 273.
*Idem* AND SMITH, J. J.—(1937) *Ibid.*, **66**, 291.
PRICE, D.—(1941) *Physiol. Zool.*, **14**, 145.
ROUS, P., AND SMITH, W. E.—(1945) *J. exp. Med.*, **81**, 597.
SCHENKEN, J., BURNS, E. L., AND KAHLE, P. J.—(1942) *J. Urol.*, **48**, 99.
WILLIS, R. A.—(1948) 'Pathology of Tumours.' London (Butterworth), p. 587.